# Key Drivers of Activity and Selectivity in Cu‐Based Catalysts for Methanol Synthesis From CO_2_: Insights From Atomically Dispersed Promoters

**DOI:** 10.1002/anie.2693293

**Published:** 2026-08-05

**Authors:** Nat Phongprueksathat, Gina Noh, Scott R. Docherty, Damian Vico van Berkel, Yannik Stiefel, Christophe Copéret, Atsushi Urakawa

**Affiliations:** ^1^ Catalysis Engineering Department of Chemical Engineering Delft University of Technology Delft The Netherlands; ^2^ Catalysis Engineering Van ’t Hoff Institute for Molecular Sciences University of Amsterdam Amsterdam The Netherlands; ^3^ Department of Chemistry and Applied Biosciences ETH Zürich Zurich Switzerland; ^4^ Department of Chemical Engineering The Pennsylvania State University Pennsylvania Pennsylvania USA; ^5^ Material Science Department of Chemistry Columbia University New York New York USA

**Keywords:** CO_2_ hydrogenation, copper, formate spillover, methanol, *operando* DRIFTS, reaction mechanism, Sabatier principle, structure–activity relationship

## Abstract

The synergistic Cu–metal oxide (Cu–MO_x_) interface is critical for selective CO_2_ hydrogenation to methanol, yet its mechanistic function, the central, long‐debated feature of industrial Cu/ZnO/Al_2_O_3_, remains ambiguous. Using *operando* transient DRIFTS‐SSITKA, we elucidate the roles of M^+^ sites (Zn^2+^, Ga^3+^, and In^3+^) on model Cu─M/SiO_2_ catalysts prepared by surface organometallic chemistry (SOMC). X‐ray absorption spectroscopy reveals that these promoters restructure from alloys after reduction to cationic species at the interface under reaction conditions. We find the promoter's electronic effect on formate bond strengths provides quantitative descriptors for both activity and selectivity. All catalysts follow a common formate spillover mechanism, with methoxy hydrogenation/desorption as the rate‐limiting step. The intrinsic CH_3_OH formation rate follows a Sabatier‐type volcano with C─O bond strength, while selectivity correlates linearly with C─H bond strength (a proxy for the formate decomposition barrier). Cu─Ga/SiO_2_ shows the fastest spillover, suppressing CO formation and yielding the highest selectivity; Cu─Zn/SiO_2_ has optimal binding for the highest activity; In^3+^ binds formate too strongly, creating a kinetic trap on the strong‐binding side of the volcano. These findings bring mechanistic clarity to the debated Cu─Zn(O) synergy, showing selectivity is governed by a balance of electronic stabilization and spillover dynamics.

## Graphical abstract

Mechanistic studies on model Cu─M/SiO_2_ catalysts (M = Zn, Ga, and In) reveal that promoters enable a formate spillover pathway from Cu to M^n^^+^ (n = 2 or 3) sites. The promoter's electronic effect on formate bond strengths serves as a quantitative descriptor for activity and selectivity, balancing spillover dynamics with intermediate stabilization to suppress CO formation and drive methanol synthesis.

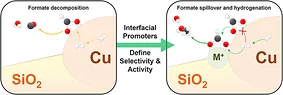

## 1 Introduction

One promising strategy to mitigate greenhouse gas emissions and combat global warming is the conversion of CO_2_ into valuable products using green (e.g., produced through water electrolysis sourced by renewable energies) or blue H_2_. Among such chemicals, methanol is positioned as the most versatile chemical feedstock and energy carrier toward a fossil‐fuel–free economy, known as the “methanol economy” [1].

Methanol synthesis from syngas (CO and H_2_) in the presence of a small amount of CO_2_ is a commercially established technology, catalyzed by Cu/ZnO/Al_2_O_3_ [2]. While the Cu/ZnO/Al_2_O_3_ catalyst is widely used in industrial methanol synthesis due to its balance of activity, stability, and selectivity, the precise nature of its active sites and the reaction mechanism remain subjects of ongoing debate, particularly in the context of CO_2_ hydrogenation [3]. The Cu/ZnO/Al_2_O_3_ catalyst is dynamic and can change its structure [4]. During reductive catalyst activation, Cu─Zn alloy is formed via migration of ZnO_x_ onto Cu^0^ [5]. Under CO_2_ hydrogenation conditions, CO_2_ oxidizes Zn, leading to Cu^0^ particles that are in contact with ZnO_x_ [6]. Such structural changes are proposed to increase the activity by generating adsorption sites for oxygen‐containing intermediates (e.g., formate). Further migration of Zn forms metastable ZnO_x_ overlayer on the Cu surface [7]. This critical ZnO─Cu interface, which facilitates methanol formation via a formate pathway, is also reported to form in situ from the surface oxidation of Cu─Zn alloys under reaction conditions [8]. This interdependent structural relationship, which dictates catalytic performance, is established by two pivotal events: the reduction of CuO and the subsequent ripening and re‐oxidation of ZnO [9].

In elucidating active sites, reaction intermediates, and reaction mechanisms during CO_2_ hydrogenation, two gaps, namely pressure and material gaps, are widely recognized, representing obstacles and complicating the establishment of structure–activity relationships [10]. The pressure gap toward industrial conditions is being addressed by clarifying pressure‐dependent active catalyst structures [11]. However, the “material gap” remains a significant impediment for mechanistic studies. Industrial Cu/ZnO/Al_2_O_3_ catalysts have an inherent complexity and active site heterogeneity, which requires the use of a model catalyst to deconvolute the crucial Cu─ZnO interaction and gain precise molecular insights [12]. This analytical challenge is compounded by the high loading of bulk Cu within the industrial Cu/ZnO/Al_2_O_3_, which strongly absorbs infrared (IR) radiation and hinders mechanistic studies using IR spectroscopy. In this regard, model Cu‐based catalysts can be prepared using surface organometallic chemistry (SOMC), which allows precise control of active sites, composition, interfacial sites, and metal‐support interactions [13]. This method allows for the creation of isolated metal sites on support, for example, SiO_2_, while removing interference from the bulk properties of metal oxides. Importantly, these isolated M^+^ sites are distinct from bulk oxides such as ZnO, In_2_O_3_, or ZrO_2_, which can hydrogenate CO_2_ to methanol in their own right via cooperative surface chemistry (e.g., oxygen vacancies, extended acid–base ensembles); here, the isolated cations instead serve primarily to modify the Cu─M interface. For example, the Cu─Zn/SiO_2_ catalysts generated via SOMC with Zn^2+^ single sites provide insights into Cu─Zn alloy and Zn redox behaviors without the contribution of bulk ZnO; extrapolation of the information can provide useful insights into Cu/ZnO/Al_2_O_3_ systems [14].

During CO_2_ hydrogenation over Cu─ZnO‐based catalysts, there is a general consensus that formate is the key surface intermediate that is further hydrogenated to methanol [8]. However, formate intermediates were not observed ex situ with IR and/or solid‐state nuclear magnetic resonance (NMR) analyses of several related SOMC‐derived catalysts, including Cu─Zn/SiO_2,_ Cu─Ga/SiO_2_, Pd─Ga/SiO_2_ [14, 15, 16, 17], and the Cu─In/SiO_2_ system investigated herein (Supporting Information, Figure S9), whereas their presence was confirmed over Cu─Ti/SiO_2_ and Cu─Zr/SiO_2_ [18, 19]. In contrast, *operando* diffuse reflectance infrared Fourier transform spectroscopy (DRIFTS) confirms the presence of formate intermediates under reaction conditions for Pd─Ga/SiO_2_ [16]. These findings underline the importance of *operando* techniques. Furthermore, they highlight the general lack of information regarding the state and reactivity of formate species, in particular, how they dictate the product selectivity.

Since these SOMC‐made Cu─Zn/SiO_2_ catalysts also show activity comparable to industrial systems, they provide an ideal platform for gaining mechanistic insights into the Cu─MO_x_ interface's role in CO_2_‐to‐methanol conversion. This approach also enables the systematic study of other promoter effects, allowing a direct comparison of different single‐site promoters by ensuring uniform Cu particle size, metal dispersion, and total surface area [13].

In this study, we shed light on reactive surface species by *operando* DRIFTS to elucidate the nature of the active and selective sites for CO_2_ hydrogenation to methanol over the Cu‐based catalysts with/without single‐site promoters (Cu/SiO_2_ and Cu─M/SiO_2_, M = Zn, Ga, and In) prepared via SOMC [14, 15, 16, 17]. This approach enables the study of the roles of each promoter (M) close to the Cu nanoparticle (e.g., the Cu─Zn/SiO_2_ structure is illustrated in Figure 1).

**FIGURE 1:**
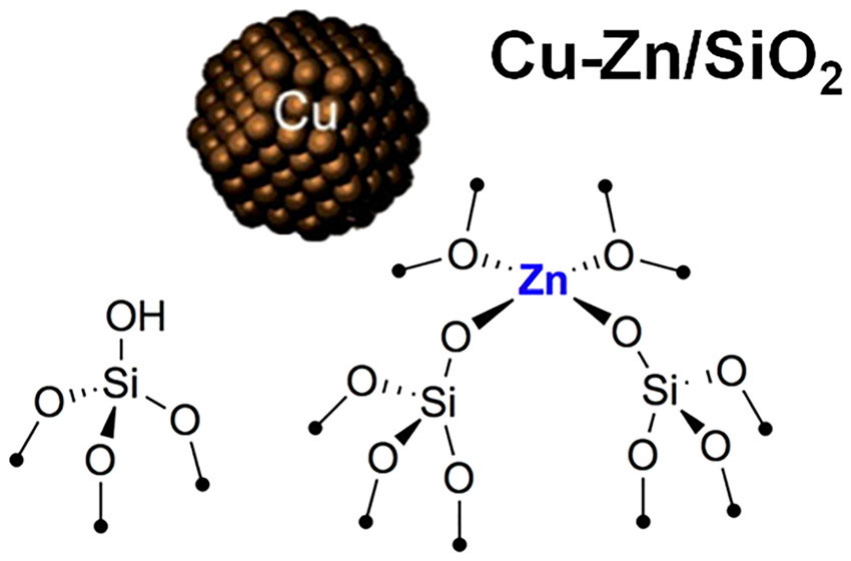
Structure of Cu─Zn/SiO_2_ prepared via SOMC reported elsewhere [14].

Transient methods in combination with the use of isotopologues, such as steady‐state isotopic transient kinetic analysis (SSITKA), clarified that the structure and thus stability of surface formate species are affected by the nature of the interfacial sites. This explains how the reaction pathway is altered toward desired methanol or undesired carbon monoxide by an effective promoter, clarifying a widely debated yet important mechanistic aspect.

## 2 Results and Discussion

### 2.1 What Is the Impact of Single‐Site Promoters on Catalytic Activity and Selectivity?

The series of catalysts, consisting of Cu nanoparticles supported on SiO_2_ decorated with isolated promoter sites (Cu─M/SiO_2_, where M = Zn, Ga, Zr, or In) or on bare SiO_2_ (Cu/SiO_2_), see Figures S5 and S6 for synthesis and characterization details and Table S4 for the Cu loadings, Cu particle sizes (measured from transmission electron microscopy, TEM), metal dispersions (N_2_O chemisorption), and BET surface areas. All catalysts were evaluated for catalytic performance under identical CO_2_ hydrogenation conditions as shown in Figure 2. Noteworthily, the isolated promoter sites are not catalytically active on their own: under identical reaction conditions, M/SiO_2_ supports (M = Zn, Ga, Zr, and In) exhibit CO_2_ hydrogenation conversions below detection limits. This is consistent with previous reports for Zn/SiO_2_ [14], Ga/SiO_2_ [15], and Zr/SiO_2_ together with related group IV/V single sites [20], and is expected, because the isolated M cations dispersed on the otherwise innocent SiO_2_ support cannot readily dissociate both CO_2_ and H_2_, a step that requires the Cu nanoparticles (*vide infra*).

**FIGURE 2:**
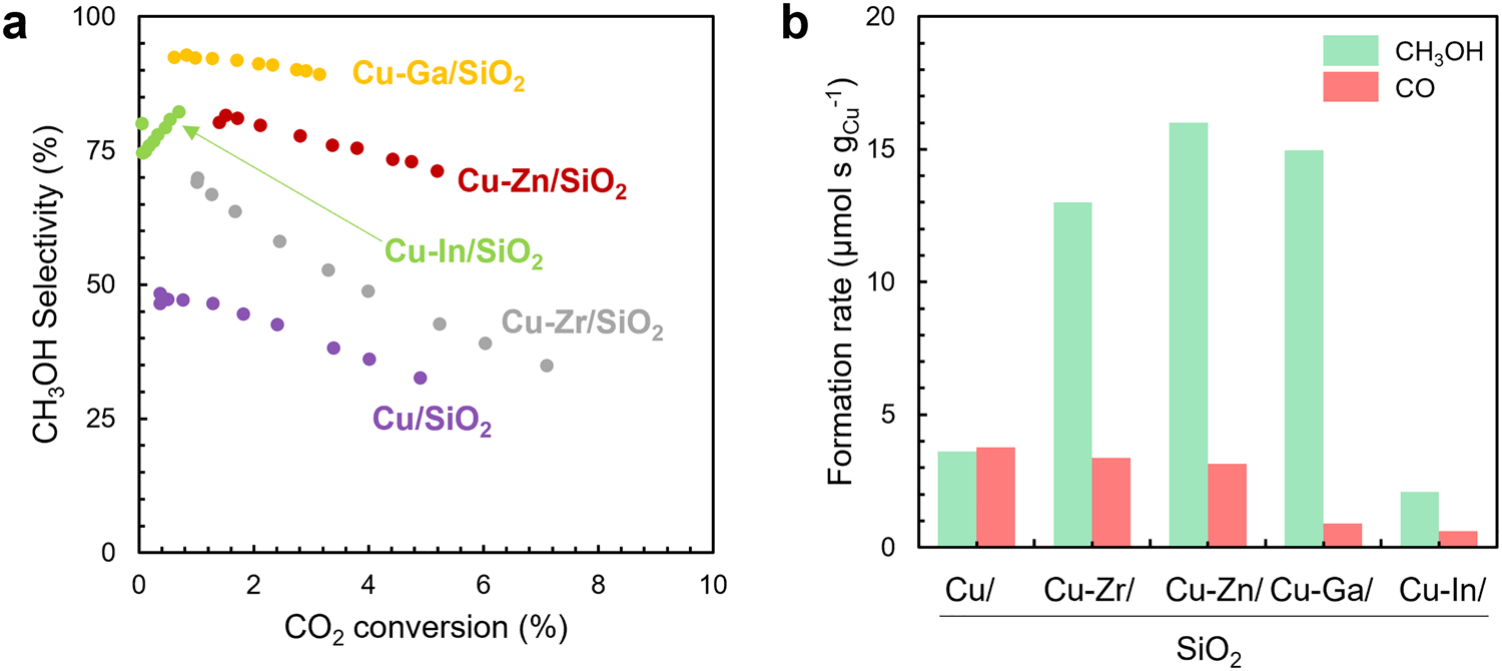
Catalytic activity of Cu/SiO_2_, Cu─Zr/SiO_2_, Cu─Zn/SiO_2_, Cu─Ga/SiO_2_, and Cu─In/SiO_2_: (a) overall CH_3_OH selectivity and CO_2_ conversion and (b) Intrinsic formation rates for CH_3_OH and CO. Pretreatment was performed at 300°C under 20 NmL min^−1^ of H_2_ for 2 h. Reaction conditions are ca. 250 mg catalyst mixed with 5 g of SiC, 230°C, 25 bar, H_2_/CO_2_ = 3, total flow rate 6–100 NmL min^−1^, TOS = 180 min.

The overall methanol selectivity shows a strong dependence on the single‐site promoters. As shown in Figure 2a, all promoted catalysts exhibit significantly higher methanol selectivity (>70%) compared to Cu/SiO_2_ (48%) at low conversions (<1%). Notably, Cu─Ga/SiO_2_ maintains exceptionally high selectivity (89%–92%) even as conversion approaches 3–4%, outperforming Cu─Zn/SiO_2_ (71%–80%), Cu─In/SiO_2_ (75%–82%), and Cu─Zr/SiO_2_ (36%–69%), all of which show a more pronounced decline with conversion. Cu/SiO_2_ exhibits the poorest selectivity (below 40%) at higher conversions, clearly highlighting the role of promoters. Notably, CH_4_ and DME were below detection limits, and the only carbon‐containing by‐product is CO. Its selectivity ranges from ∼8%–11% over Cu─Ga/SiO_2_ to >50% over Cu/SiO_2_ at comparable conversions; the complete dataset of CO_2_ conversions, CH_3_OH and CO selectivity is compiled in Table S2, and the corresponding intrinsic formation rates in Table S3.

Interestingly, a different trend emerges when examining the intrinsic formation rates (Figure 2b). Because quantifying the dynamically changing, dual‐site active atoms is inaccurate for these alloys (*vide infra*), these rates are normalized by copper mass (µmol s^−1^ g_Cu_^−1^) to ensure a fair comparison across the catalysts. Cu─Zn/SiO_2_ displays the highest intrinsic activity toward methanol surpassing Cu─Ga/SiO_2_ and Cu─Zr/SiO_2_. Both are significantly more active than the unpromoted Cu/SiO_2_. In contrast, Cu─In/SiO_2_ exhibits lower intrinsic methanol formation rates than even the unpromoted catalyst under these conditions. The selectivity trends are further clarified by the intrinsic CO formation rates (Figure 2b). Cu/SiO_2_, Cu─Zn/SiO_2_, and Cu─Zr/SiO_2_ exhibit similar, relatively high CO rates. In stark contrast, both Cu─Ga/SiO_2_ and Cu─In/SiO_2_ demonstrate significant suppression of the CO formation pathway. These catalytic results highlight distinct roles for each promoter compared to Cu/SiO_2_: Zn^2+^ and Zr^4+^ primarily boost the intrinsic rate of methanol formation without suppressing CO; Ga^3+^ strongly suppresses CO while maintaining high methanol activity, leading to superior overall selectivity; In^3+^ also suppresses CO but at the cost of significantly reduced methanol activity. Understanding the origins of these pronounced differences in activity and selectivity requires a detailed investigation of the surface intermediates and reaction pathways, which will be explored in the following sections.

### 2.2 What Is the Structural Nature of the Promoter under Reaction Conditions?

The promotional role of isolated, Lewis acid sites, such as Zr^4+^ sites at the Cu interface in Cu─Zr/SiO_2_ prepared via SOMC remains in their oxidation state after reduction and under reaction conditions, giving this well‐established baseline for a nonreducible promoter [19]. The present study focuses primarily on comparing the effects of Zn^2+^, Ga^3+^, and In^3+^ promoters, which exhibit more dynamic structural and redox behavior.

Catalysts prepared with Zn^2+^ or Ga^3+^ initially form CuZn or CuGa alloy nanoparticles upon reduction, alongside residual Zn^2+^ or Ga^3+^ sites. Crucially, in situ X‐ray absorption spectroscopy (XAS) showed that these alloys undergo de‐alloying under CO_2_ hydrogenation conditions [14, 15, 16, 17]. For Cu─Ga/SiO_2_, this resulted in complete re‐oxidation to form Cu^0^ nanoparticles interfaced with Ga^3+^ sites [15], while for Cu─Zn/SiO_2_, the de‐alloying was partial, leading to a dynamic mixture of Cu^0^, isolated Zn^2+^ sites, and some remaining reduced Zn^0^ species under reaction conditions [14]. XAS analysis of the Cu─In/SiO_2_ catalyst reveals analogous behavior. In situ Cu K‐edge near edge spectra (XANES; Figure 3a, see Figure S1 for experimental procedure) indicated that Cu was present primarily as metallic Cu^0^ after pretreatment with H_2_ and remained so under CO_2_ hydrogenation conditions, similar to observations for related Cu─M/SiO_2_ systems. In addition, *ex situ* In K‐edge XANES spectra (Figure 3b) of the reduced catalyst showed a significant shift in the absorption edge energy relative to the In_2_O_3_ reference, consistent with the formation of reduced In species. Fine structure (EXAFS) analysis confirms the formation of a Cu─In_x_ alloy: a simultaneous fit of the Cu and In K‐edges of the reduced catalyst reveals a Cu─In/In─Cu scattering path at 2.69 Å, with a contraction of the Cu─Cu distance to 2.475 Å relative to fcc Cu (2.55 Å), consistent with substitutional incorporation of In into the Cu nanoparticles (Figure S7 and Table S5). The In K‐edge fit further shows that In is present in two co‐existing environments: an oxygen‐coordinated In^3+^ environment comparable to the In/SiO_2_ precursor and a metallic In environment from which roughly one‐third of the In is estimated to be alloyed in the as‐prepared catalyst, the remainder persisting as isolated In^3+^ sites (see Supporting Information, Section 2.2.3). Upon exposure to reaction conditions, the edge energy of the ex situ reacted sample shifts back to values indicative of In^3+^, suggesting that the Cu─In/SiO_2_ system also undergoes re‐oxidation and de‐alloying (Figure S8).

**FIGURE 3:**
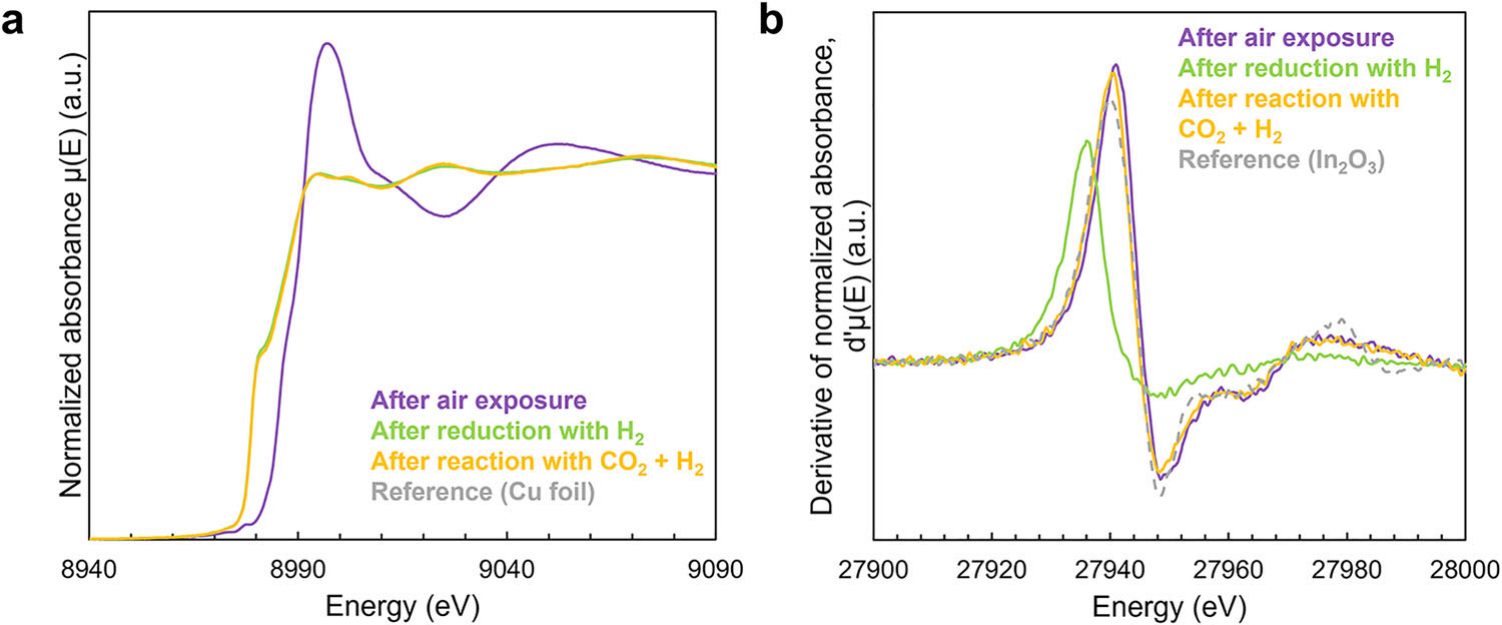
*Operando* XAS spectra of the Cu─In/SiO_2_ catalyst. (a) In situ Cu K‐edge XANES spectra recorded after air exposure (purple), after reduction with H_2_ (green), and under reaction with CO_2_ + H_2_ (yellow). The spectrum of a Cu foil is included as a reference (dashed grey). (b) Ex situ In K‐edge first derivative XANES spectra recorded after air exposure (purple), after reduction with H_2_ (green), and after reaction with CO_2_ + H_2_ (yellow). The spectrum of In_2_O_3_ is included as a reference (dashed grey). Pretreatment was performed at 300°C under 10 NmL min^−1^ of 20% H_2_ for 1 h. Reaction conditions are ca. 10 mg catalyst, 230°C, 5 bar, H_2_/CO_2_ = 3, total flow rate 10 NmL min^−1^, TOS = 60 min.

Therefore, Zn^2+^, Ga^3+^, and In^3+^ promoters, prepared via SOMC, all share characteristic dynamic restructuring involving dopant redox, differentiating them from isolated Zr^4+^ promoters. The alloyed fraction of the promoter in the as‐prepared (reduced) state is comparable across the three systems (≈25%–50% for In, Ga, and Zn; Table S6), while the extent of re‐oxidation under reaction conditions differs: it is total for Ga^3+^ and In^3+^, but only partial for Zn^2+^ [21]. This in situ generation of interfacial M^+^ sites via de‐alloying represents a fundamentally different scenario compared to the static Zr^4+^ sites. This dynamic nature is thought to be the key to their distinct activity and selectivity, as it potentially creates a high density of active interfacial sites in proximity to the Cu nanoparticles. However, the atmosphere‐dependent generation of interfacial M^+^ sites means their density under reaction conditions is not directly accessible by ex situ characterization (e.g., N_2_O chemisorption). Consequently, catalytic rates are compared on a per‐Cu‐mass basis rather than as site‐based turnover frequencies (TOFs) (*vide supra*). Importantly, this re‐oxidized, cationic state is the one present during formate binding: under the same reaction atmosphere, *κ^2^*‐HCOO* forms on both Cu and the promoter (*vide infra*). It is therefore the oxidized, Lewis‐acidic M^+^ site, not a reduced or alloyed form, that stabilizes the formate intermediate.

### 2.3 Surface Intermediates in CO_2_ Hydrogenation

The surface intermediates formed during the CO_2_ hydrogenation over Cu/SiO_2_, Cu─In/SiO_2_, Cu─Ga/SiO_2_, and Cu─Zn/SiO_2_ catalysts were investigated by *operando* DRIFTS (Figure 4a), after the pretreatment in H_2_ (followed by purging with He, see Figure S2 for experimental procedure). The temporal evolution of surface species was monitored after switching to the reaction mixture of H_2_/CO_2_. The assignments of the IR bands are listed in Table S1. Upon passing the reaction mixture, bidentate formate on Cu (*κ* ^2^‐HCOO* (Cu)) at 2856 and 2931 cm^−1^ was the first and most common surface species detected over all catalysts. Moreover, *κ^2^*‐HCOO* (Cu) appeared simultaneously with bidentate formate on Zn^2+^ (*κ* ^2^‐HCOO* (Zn^2+^)) over Cu─Zn/SiO_2_ at 2896 cm^−1^. The time‐resolved *operando* DRIFT spectra of Cu/SiO_2_, Cu─In/SiO_2_, and Cu─Ga/SiO_2_ catalysts are similar (Figure S10). Interestingly, while *κ* ^2^‐HCOO* was clearly observed on Zn^2+^, its presence on Ga^3+^ or In^3+^ could not be confirmed firmly by this approach alone (without isotopic and transient experiments). After 90 min, methoxy on SiO_2_ CH_3_O*(SiO_2_) at 2858 cm^−1^ and surface‐bound methanol (CH_3_OH*) at 2981 cm^−1^ built up slowly on the catalysts (Figure 4c). The delay in mass spectrometry response of CH_3_OH compared to that of H_2_O (Figure 4b) hinted at a stronger interaction of CH_3_OH with SiO_2_ than that of H_2_O, for example, by the adsorption of CH_3_OH product over Si─OH transforming into Si─O─CH_3_ (i.e., methoxy or CH_3_O*(SiO_2_)). It should be noted that carbonyl species (CO*) were not detected over any of the catalysts studied.

**FIGURE 4:**
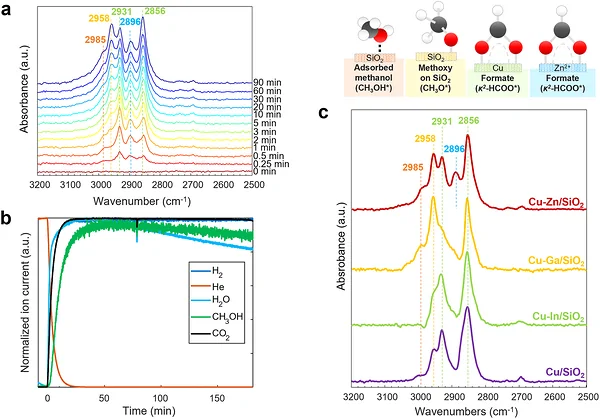
(a) Time‐resolved *operando* DRIFT spectra of the surface species formed during the CO_2_ hydrogenation reaction over pre‐reduced Cu─Zn/SiO_2_ catalysts and structures of relevant surface species. (b) Corresponding normalized ion current signal obtained from mass spectrometry. (c) *Operando* DRIFT spectra of the surface species formed during the CO_2_ hydrogenation reaction over Cu/SiO_2_, Cu─In/SiO_2_, Cu─Ga/SiO_2_, and Cu─Zn/SiO_2_ catalysts after reaching steady state (2 h). Pretreatment was performed at 300°C under 10 NmL min^−1^ of H_2_ for 2 h. Reaction conditions are 10 mg catalyst, 230°C, 10 bar, H_2_/CO_2_ = 3, total flow rate of 10 NmL min^−1^.

The detection of *κ* ^2^‐HCOO* (Cu), particularly in the presence of promoters such as Zn^2+^, Ga^3+^, or In^3+^, was initially unexpected since the *κ* ^2^‐HCOO* (Cu) was unobservable and represented by only *κ* ^2^‐HCOO* (Zn^2+^) in our previous study where the Cu‐Zn/SiO_2_ was prepared via SOMC and atomic layer deposition (ALD) [17]. In the current study, the observation of both *κ* ^2^‐HCOO* (Cu) and *κ* ^2^‐HCOO* (Zn^2+^) as kinetically distinguishable species (*vide infra*) help understand the interplay between these formate species—on Cu and promoter sites—may critically influence CH_3_OH selectivity as discussed later.

### 2.4 Relationship between Formate and Methoxy Surface Species and Methanol Formation Rate

Transient isotopic labeling experiments are powerful methodologies that can provide insights into active species with minimal perturbation to the steady state [22]. As shown in Figure 5 after switching from H_2_+CO_2_ to D_2_+CO_2_ and vice versa (see Figure S3 for experimental procedure), *κ^2^*‐HCOO* (Cu) at 2931 and 2850 cm^−1^ and *κ^2^*‐HCOO* (Zn^2+^) at 2896 cm^−1^ exchanged instantaneously to *κ^2^*‐DCOO*(Cu) at 2167 cm^−1^ and *κ^2^*‐DCOO*(Zn^2+^) at 2184 cm^−1^, while CH_3_O*(SiO_2_, 2958 and 2858 cm^−1^) and CD_3_O*(SiO_2_, 2080 cm^−1^) exchanged slowly. This exchange simplifies the spectra, as C─H combination bands (e.g., *ν*(C─O) + *δ*(C─H) at 2931 cm^−1^) shift out of this region, leaving only the fundamental *ν*(C─D) stretches at 2167 cm^−1^. For methoxy, the symmetric *ν*_s_(C─H) stretch at 2858 cm^−1^ shifts down to the observed *ν*_s_(C─D) band at 2080 cm^−1^, while the asymmetric *ν*_as_(C─H) stretch at 2958 cm^−1^ is expected to shift to ∼2160 cm^−1^, where it overlaps with the intense deuterated formate bands (see Supporting Information for calculations of C─D stretching frequencies).

**FIGURE 5:**
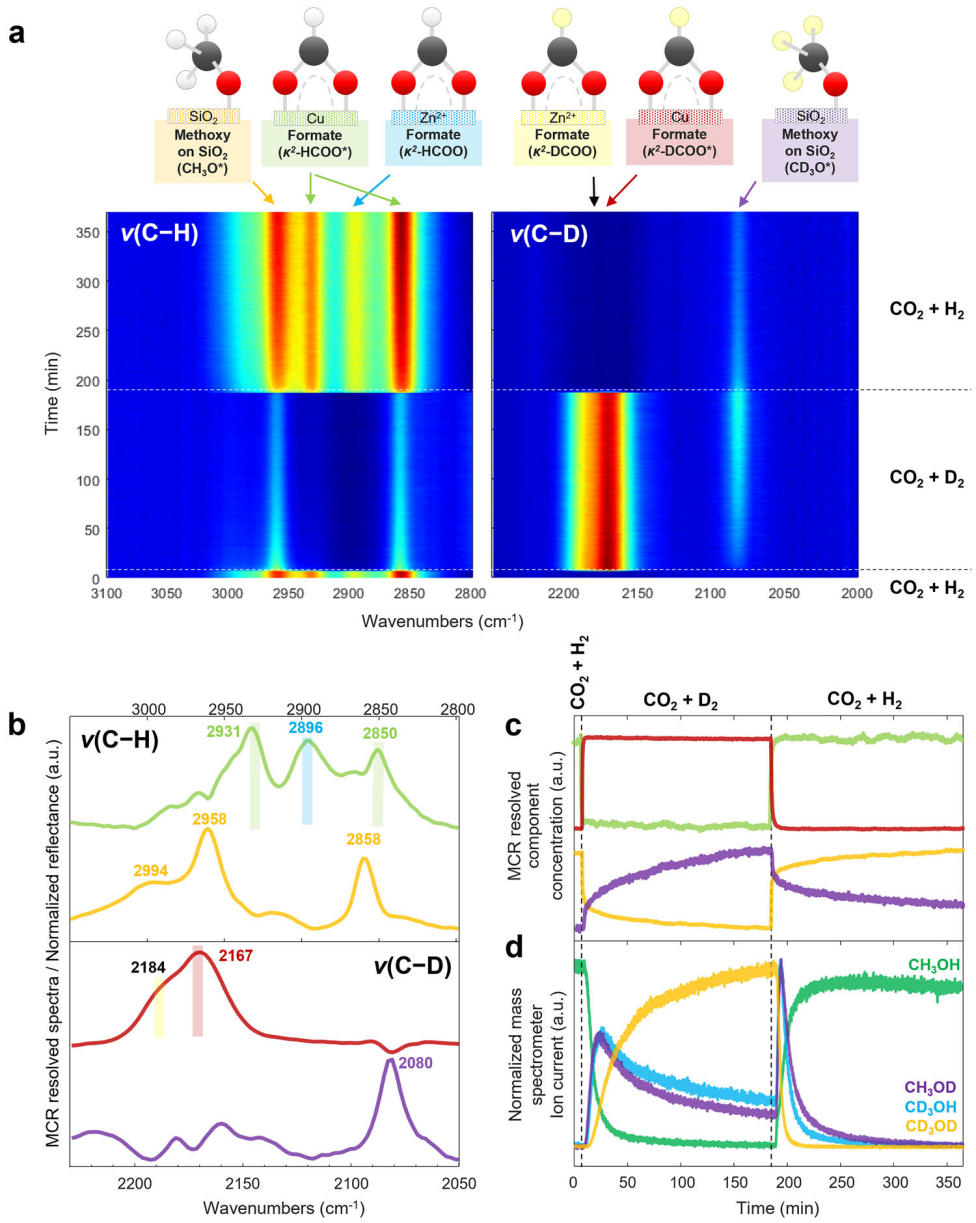
Transient responses of surface species and gas products during the steady‐state isotopic switching from CO_2_ + D_2_ to CO_2_ + H_2_ over Cu─Zn/SiO_2_ catalysts. (a) Time‐resolved DRIFT spectra of *ν*(C─H) region and *ν*(C─D) region. (b) Component's spectra obtained by MCR applied on the time‐resolved DRIFT spectra. (c) Concentration profiles of the spectra of the corresponding components obtained by MCR. (d) Corresponding normalized ion current signal of isotope‐labeled products. Reaction conditions: 10 mg catalyst, H_2_(or D_2_)/CO_2_ = 3, T = 230°C, P = 10 bar, F_total_ = 10 NmL min^−1^.

The separation of kinetically unique spectral features of the surface species during the SSITKA experiment was obtained using multivariate curve resolution (MCR), enabling blind‐source separation (i.e., without references) [23, 24]. As shown in Figure 5b, MCR analysis of the time‐resolved spectra (Figure 5a) yielded deconvoluted spectra of kinetically distinct species (Figure 5b) and their concentration profiles (Figure 5c). The normalized gas concentration profiles determined by mass spectrometry during the transient study are presented in Figure 5d. The kinetics of formation and hydrogenation of the *κ^2^*‐HCOO* (Zn^2+^) were indistinguishable from *κ^2^*‐HCOO* (Cu) with the time resolution of this study (10 s). The fast decay responses of surface *κ^2^*‐HCOO* (Cu and Zn^2+^) and the concomitant increase of *κ^2^*‐DCOO* species indicate that kinetic isotope effects (KIE) are negligible. This may also imply that these formates are easily hydrogenated to CH_3_OH and are not spectators in CO_2_ hydrogenation. The isotopic exchange of *κ^2^*‐HCOO*/*κ* ^2^‐DCOO* species was almost instantaneous (Figure 5c, red and green lines), suggesting that H_2_/D_2_ dissociative adsorption and formate formation were fast, and not the rate‐limiting step in methanol formation [25].

On the other hand, prominent KIE was observed for methanol formation (Figure 5d, green line (CH_3_OH, upon switching to H_2_+CO_2_) vs. yellow line (CD_3_OD, upon switching to D_2_+CO_2_)), showing significant delay in the formation of CD_3_OD compared to that of CH_3_OH. This suggests the presence of KIE for formate hydrogenation to CH_3_OH and/or for CH_3_O* activation by H addition/activation toward formation/desorption of CH_3_OH.

The formation profiles of the transiently produced methanol species (CH_3_OD vs. CD_3_OH, Figure 5d) after the isotopologue switch are convenient reaction‐rate indicators of formate hydrogenation (C─H/C─D formation) or activating methoxy species (O─H/O─D formation) that dominate before the switch. To determine the rate‐limiting step, we analyzed the products formed immediately after the isotopic switch from CO_2_​+D_2_​ to CO_2_​+H_2_​ (Figures 5d and S11a). The formation of CD_3_​​OH arises from the hydrogenation of pre‐existing deuterated surface species (like CD_3_​O*(SiO_2_​)), while the formation of CH_3_​​OD requires the full hydrogenation of newly adsorbed carbon with incoming H_2_. Crucially, the formation of both CD_3_​​OH and CH_3_​​OD were observed to be very similar. If C─H bond formation was the rate‐determining step, the rate for CH_3_​​OD would be substantially lower than for CD_3_​​OH. The comparable rates therefore indicate that the KIE for carbon hydrogenation is small and that these steps are not rate‐limiting. This suggests that a different step, such as the methanol formation or desorption, controls the overall reaction rate. The reverse experiment, switching from CO_2_​+H_2_​​ to CO_2_​+D_2_​​, yielded analogous results, supporting this conclusion (Figures 5d and S11b). More details on the transient responses and steady‐state distribution of isotopologues are summarized in Figure S13).

Having established that the initial carbon hydrogenation steps are rapid, the rate‐limiting step can be identified from the spectroscopic data. A slow isotopic exchange response for the surface methoxy species (CH_3_​O*(SiO_2_​), or CD_3_​O*(SiO_2_​)), correlates directly with the delayed formation of gas‐phase methanol (CH_3​_OH or CD_3_OD) observed by mass spectrometry (Figures 5c and 5d). This strong correlation, along with a significant kinetic isotope effect for this process (KIE = 2.78, Figure S12), led to the conclusion that the final hydrogenation and/or desorption of the methoxy species is the rate‐limiting step for CO_2_​ hydrogenation over Cu‐Zn/SiO_2_. Further analysis of the MCR‐resolved responses reveals two surface methoxy populations: a rapidly exchanging reactive pool, likely in proximity to the Cu/Zn^2+^ interface, that governs both the observed KIE and the rate‐limiting behavior, as well as a slowly‐exchanging spectator pool tentatively assigned to remote SiO_2_ sites (Figure S14). This insight refines our previous understanding of this system. While we previously considered the CH_3​_O*(SiO_2_​) species to be a stable, non‐participatory spectator [17], this work reveals its crucial role as a key intermediate participating directly in the rate‐determining step of the catalytic cycle, an insight made possible by isotopic transient analysis.

Notably, SSITKA experiments on Cu/SiO_2_, Cu─Ga/SiO_2_, and Cu─In/SiO_2_ showed similar responses of surface species and isotopic methanol products, suggesting a common reaction mechanism via formate hydrogenation for all catalysts (Figures S15–S17). However, the intriguing details lay in the kinetically resolved spectra after MCR analysis. Similar to Cu─Zn/SiO_2_, the kinetics of *κ^2^*‐HCOO* (Ga^3+^) are indistinguishable from *κ^2^*‐HCOO* (Cu) (i.e., they appear in the same MCR‐resolved spectrum since they are not kinetically discernible, Figure S16) and suggest a similar role of Ga^3+^ to Zn^2+^.

On the other hand, *κ^2^*‐HCOO*(In^3+^) was not observed after MCR analysis (Figure S17). While confirming a common mechanism, this MCR analysis is unable to resolve the ambiguity for the Cu─In/SiO_2_ system: it cannot be distinguished whether the *κ^2^*‐HCOO*(In^3+^) formate is unobservable because it is consumed rapidly or because it is not formed in the first place. However, the electronic properties of each promoter directly influence the bond characteristics of the formate intermediates, which will be discussed in the next section.

### 2.5 Effect of Neighboring Promoter on Formate Species and Methanol Formation Over Cu‐Based Catalysts

The application of MCR also allowed for the precise isolation of component spectra, providing insights into the effects of single‐site promoters near Cu nanoparticles. To avoid interference with methoxy bands, which can complicate the spectra, the *ν*(C─D) stretching frequencies were specifically analyzed. This approach provided clear insights into the bond characteristics of formates on Cu─Zn/SiO_2_, revealing distinct vibrational frequencies at 2184 and 2167 cm^−1^ for *κ^2^*‐DCOO* (Zn^2+^) and *κ^2^*‐DCOO* (Cu) (Figure 5b), and provides direct spectroscopic evidence for the dual role of the Zn^2+^ promoter: it not only provides a distinct Lewis acid site for formate stabilization but also electronically influences the formate bound to the adjacent Cu surface.

For the other promoters (Ga^3+^ and In^3+^), distinct bands for promoter‐bound formate were not resolved in the *ν*(C–D) region, possibly due to spectral overlap with the *κ^2^*‐DCOO* (Cu) band or a low steady‐state concentration. Nonetheless, the electronic influence of all promoters on the overall formate population is evident. As summarized in Figure 6, the introduction of any single‐site promoter (Zn^2+^, Ga^3+^, or In^3+^) leads to observable shifts in the vibrational frequencies of both the C─D and C─O bonds of the detected formate species compared to Cu/SiO_2_. Since higher vibrational frequencies correspond to a shorter and stronger bond, these shifts indicate that all promoters exert a similar electronic effect, which weakens the C─O bond while strengthening the C─H bond of the surface formate.

**FIGURE 6:**
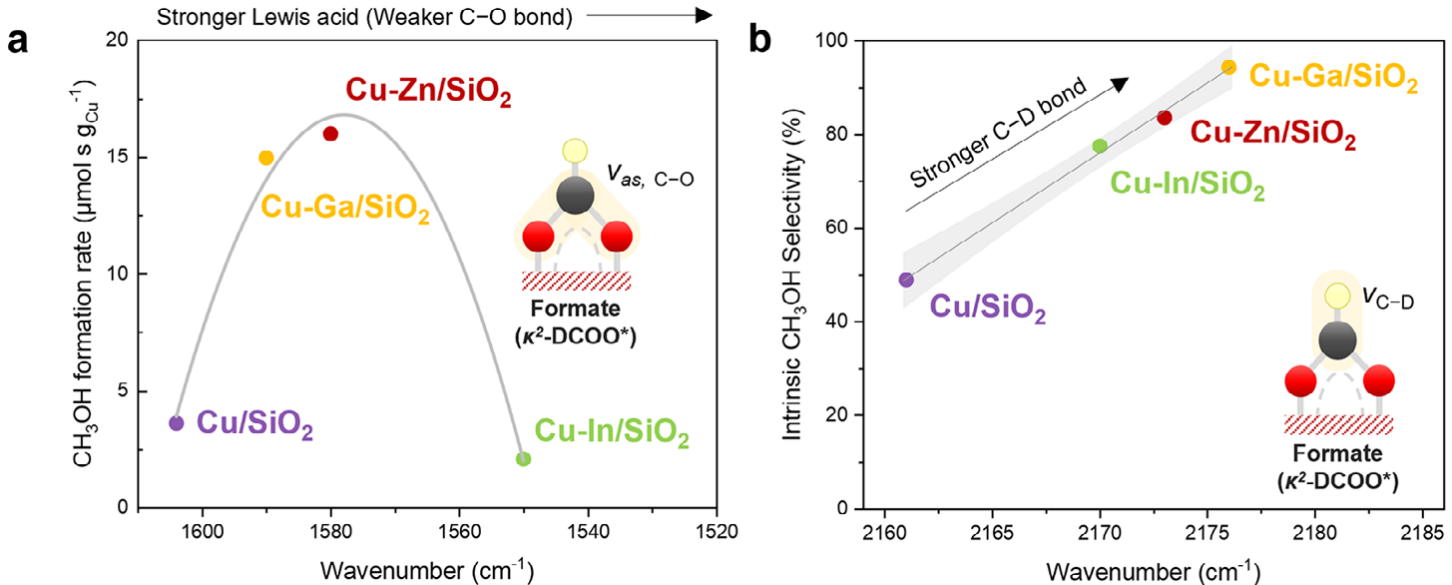
Catalytic performance of Cu/SiO_2_, Cu─Zn/SiO_2_, Cu─Ga/SiO_2_, and Cu─In/SiO_2_ catalysts as a function of formate intermediate bond strengths obtained from the isotopic transient experiment. (a) The intrinsic CH_3_OH formation rate versus the *ν*(C─O) stretching frequency, which serves as a proxy for the formate‐promoter binding strength and strength of the Lewis acid site. (b) The intrinsic CH_3_OH selectivity versus the *ν*(C─D) stretching frequency of the *κ^2^*‐DCOO* species. The *κ^2^*‐DCOO* species wavenumber for Cu─Zn/SiO_2_ (2173 cm^−^^1^) represents the band center of the overlapping *κ^2^*‐DCOO* (Cu) and *κ^2^*‐DCOO*(Zn^2+^) components (see Figure 5b (bottom)), which was determined by Gaussian fitting.

This electronic influence on the formate bonds provides the key to understanding the catalytic performance. The *ν*(C─O) stretching frequency, which acts as a proxy for the formate binding strength (and Lewis acid strength), exhibits a distinct volcano‐shaped relationship with the intrinsic CH_3_OH formation rate (Figure 6a). This trend illustrates the Sabatier principle. At high *ν*(C─O) frequencies (weak binding), unpromoted Cu/SiO_2_ binds formate too weakly for efficient activation, resulting in a low rate. Conversely, at low *ν*(C─O) frequencies (strong binding), the strong Lewis acidity of In^3+^ causes the formate to over‐stabilize. This creates a kinetic trap that prevents subsequent hydrogenation, thus suppressing the overall reaction rate. At the optimum, Cu─Zn/SiO_2_ and Cu─Ga/SiO_2_ possess an intermediate binding strength that balances formate activation with its subsequent hydrogenation, leading to the highest intrinsic activities.

These findings are consistent with previous reports on related SOMC‐derived catalysts. For example, the enhanced CH_3_OH formation rate on Cu─M/SiO_2_ (M = Ti, Zr, Hf, Nb, and Ta) was related to the Lewis acidity of the dopant and attributed to the stabilization of HCOO* and CH_3_O* intermediates at the Cu periphery [20]. It has been demonstrated that isolated Zr^4+^ [19] and Ti^4+^ [26] sites adjacent to Cu nanoparticles—as opposed to bulk oxide properties [19] —are sufficient to enhance CH_3_OH selectivity by stabilizing these formate and methoxy intermediates [26]. Similarly, Lewis acid sites (M = Y, Sc, Zr, and Ta) over Cu─M/γ‐Al_2_O_3_ were shown to simultaneously weaken the C─O bond and strengthen the C─H bond of *κ^2^*‐HCOO* via their electron‐withdrawing character, an effect that destabilizes the *κ^2^*‐HCOO* intermediate, increases the electrophilicity (positive charge) of the central carbon, makes it more susceptible to nucleophilic attack by a hydride (H^−^) from the Cu, and eventually reduces the apparent activation energy for CH_3_OH formation [27]. However, these studies, which observed a linear increase in reactivity with Lewis's acidity, effectively mapped only the weak‐binding side of the volcano plot. Indeed, over‐stabilization of HCOO* on strongly Lewis acidic γ‐Al_2_O_3_ has been shown to be detrimental, opening a side‐reaction pathway via methyl formate (HCOOCH_3_) that decomposes to promote CO selectivity [28]. Notably, this methyl formate decarbonylation is specific to the strongly Lewis acidic γ‐Al_2_O_3_ surface [28]; on SiO_2_‐ and ZnO‐based supports the ester is instead preferentially hydrogenated back to methanol [29], so this CO‐forming route is not expected to be significant for our Cu‐M/SiO_2_ catalysts, where CO formation is instead governed by direct *κ^2^*‐HCOO* decomposition on Cu.

While the C─O bond strength dictates the overall activity according to the Sabatier principle (Figure 6a), the C─D bond strength serves as a powerful and direct descriptor for CH_3_OH selectivity. As shown in Figure 6b, the intrinsic CH_3_OH selectivity correlates directly and linearly (*R* ^2^> 0.99) with the *ν*(C─D) stretching frequency of the *κ^2^*‐DCOO* intermediate.

This provides a clear mechanistic link: *κ^2^*‐HCOO* is also the key intermediate for the CO (RWGS) pathway on Cu, and its subsequent decomposition to CO involves both C─O and C─H bond cleavage (HCOO* → CO* + OH*) [30]. Based on DFT calculations, the HCOO* based pathway for CO formation (RWGS) on Cu is kinetically favorable proceeding via HCOOH* → cis‐COOH* → CO* pathway and hydrogenation of HCOOH* to H_2_COOH* is a crucial route to CH_3_OH [31]. Therefore, strengthening the C─H bond via promoter introduction (Figure 6b) could, in principle, suppress *κ^2^*‐HCOO* decomposition pathway while facilitating CH_3_OH formation. This conclusion is strongly supported by HCOOH‐TPSR which proves that the higher stability of HCOO* can inhibit its decomposition into CO and directly correlates with higher methanol selectivity [32].

### 2.6 Role of Promoter in Driving Methanol Formation—Relation of Formate Spillover and Methanol Selectivity

The nature of the active site of Cu─ZnO‐based catalyst remains highly debated, and it was proposed that CO_2_ hydrogenation to CH_3_OH occurs via zinc formate Zn(HCOO)_2_ intermediates originating from hydrogen dissociation over Cu, H‐spillover to ZnO, and subsequent CO_2_ activation over ZnO [33]. While ZnO can generate formate directly via this route, it is associated with bulk or clustered ZnO; in our catalyst the working Zn speciation is dominated by isolated interfacial Zn^2+^ (in situ XAS), which is itself inactive for CO_2_ hydrogenation and remains fully oxidized in the absence of Cu [14], making independent CO_2_ activation at Zn^2+^ an unlikely formate source and pointing instead to formate generation on Cu followed by spillover (*vide infra*, Figure 7) [34]. Notably, *operando* studies of industrial‐type catalysts indicate that the catalytically relevant zinc under reaction conditions is not bulk ZnO but is instead oxidized, dispersed interfacial Zn. This includes a reactive zinc formate intermediate and, under high‐conversion conditions, low‐nuclearity ZnO_x_ species resembling silica‐supported Zn, consistent with the single‐site Zn^2+^ in this study [33, 35].

**FIGURE 7:**
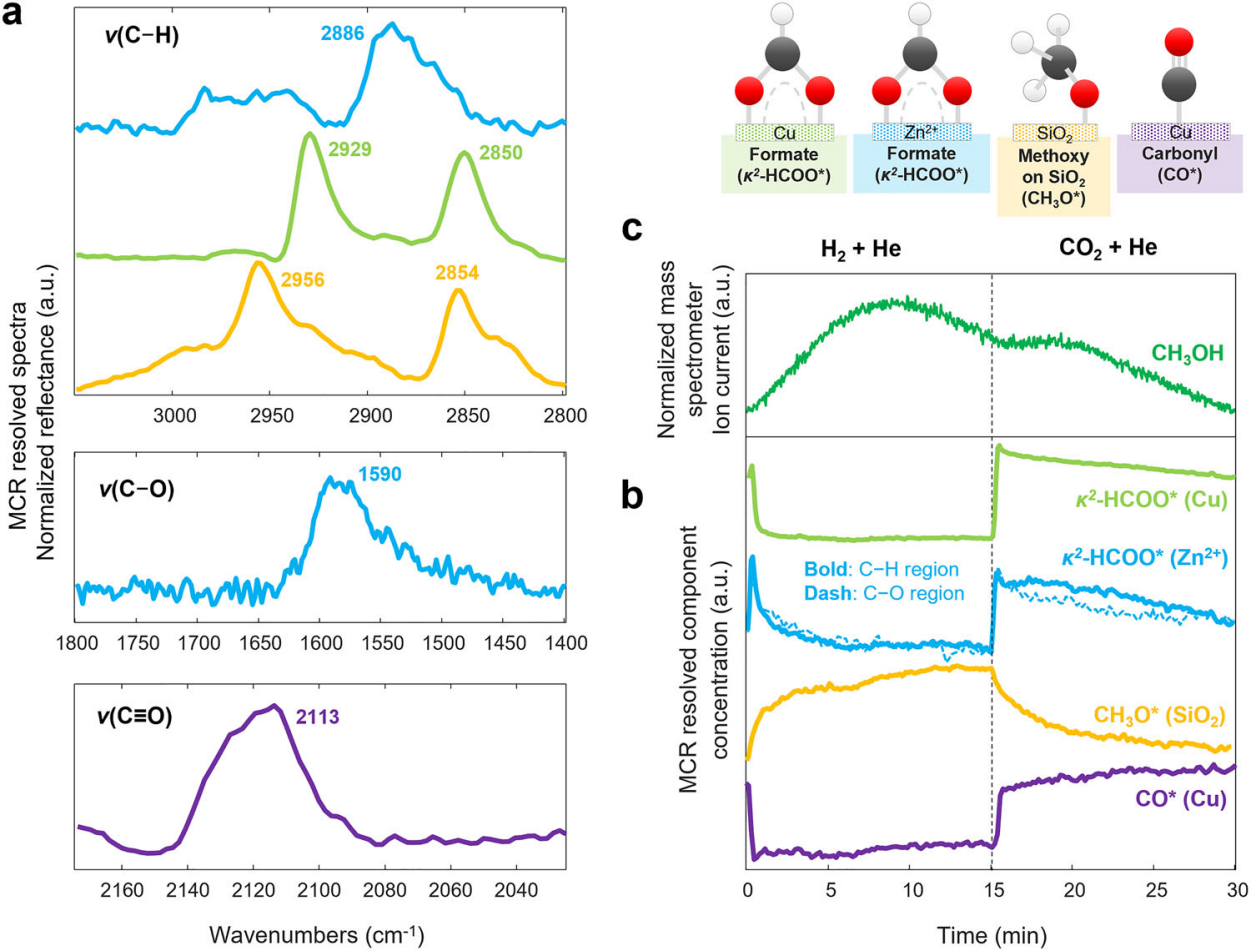
Transient responses of surface species and gas products during switching from He + CO_2_ to H_2_ + He over Cu─Zn/SiO_2_ catalysts. (a) Component spectra obtained by MCR applied on the time‐resolved DRIFT spectra. (b) Concentration profiles of the spectra of the corresponding components obtained by MCR‐ALS. Reaction conditions. (c) Corresponding normalized ion current signal of CH_3_OH products. Reaction conditions: 10 mg catalyst, He/CO_2_ = H_2_/He = 3, T = 230°C, P = 20 bar, F_total_ = 10 NmL min^−1^.

Even using a well‐defined Cu─Zn/SiO_2_ prepared via SOMC, it was also challenging to pinpoint the origin of the high CH_3_OH selectivity due to the presence of Lewis acidic Zn sites and/or reduced Zn sites (alloy) [14]. While some recent *operando* studies have concluded that formates bound directly to the metallic Cu^0^ surface are the main reaction intermediates [36], other works highlight that Lewis acidic sites at the perimeter can play an essential role as an active reservoir for *κ^2^*‐HCOO*, which can spillover from the metal particle and be stabilized [34, 37, 38]. The selectivity to CH_3_OH over CO should be related to the spillover rate toward M^+^ sites (Zn^2+^, Ga^3+,^ and In^3+^) as well as the stability of HCOO* over M^+^ sites, which can be elucidated by transient experiments.

To directly probe this proposed formate spillover pathway, a transient experiment separating the CO_2_ and H_2_ activation steps was employed (see Figure S4 for experimental procedure). By alternatingly passing CO_2_ or H_2_, this method provides further insights into CO_2_ activation and subsequent hydrogenation. The limited supply of reactants (especially H_2_) allows the capturing of the fast‐forming species (e.g., CO* or *κ^2^*‐HCOO* over Zn^2+^, Ga^3+^, In^3+^), which were readily hydrogenated during steady‐state CO_2_ hydrogenation.

The evolution of the surface species during the CO_2_ and H_2_ phases over the Cu─Zn/SiO_2_, is presented in Figure 7. Unlike the previous steady‐state experiment, this alternating‐feed method allowed the formation and consumption of *κ^2^*‐HCOO* (Cu) and *κ^2^*‐HCOO* (Zn^2+^) to be kinetically distinguished using MCR‐ALS (Figure 7a).

The resulting concentration profiles (Figure 7b) reveal a clear temporal sequence of events at the beginning of the H_2_ phase. First, *κ^2^*‐HCOO* (Cu) forms and is consumed rapidly. Second, after a slight delay, *κ^2^*‐HCOO* (Zn^2+^) forms and then begins to decay, but at a much slower rate than the *κ^2^*‐HCOO* (Cu). Finally, the decay of this *κ^2^*‐HCOO* (Zn^2+^) directly correlates with the formation of CH_3_O*(SiO_2_) and the detection of gas‐phase CH_3_OH (Figure 7c). This sequential transfer of the *κ^2^*‐HCOO* intermediate from Cu to Zn, which then leads to the final product, provides direct experimental evidence for the proposed formate spillover pathway from Cu to adjacent Zn^2+^ sites, where the intermediate is stabilized for hydrogenation to CH_3_OH.

For Cu‐Ga/SiO_2_, the MCR analysis showed kinetically indistinguishable spectra comprising *κ^2^*‐HCOO* (Cu), *κ^2^*‐HCOO* (Ga^3+^), CH_3_O*(Ga^3+^) and adsorbed CH_3_OH (Figure S20), all of which were consumed rapidly under H_2_. This suggests that the spillover of *κ^2^*‐HCOO* from Cu to Ga^3+^ and its subsequent hydrogenation are both fast, such that the intermediates cannot be resolved at this time scale (faster than that over Cu‐Zn/SiO_2_). In contrast, for Cu‐In/SiO_2_, while MCR analysis also shows evidence of spillover of kinetically indistinguishable spectra comprising *κ^2^*‐HCOO* (In^3+^) and CH_3_O*(In^3+^) (Figure S19), they were not consumed during the H_2_ phase and instead increased and reached a stable plateau. This result suggests that *κ^2^*‐HCOO* once spilled over from Cu to In^3+^ was less favorable to further hydrogenate to produce CH_3_OH, which is due to overly strong binding of *κ^2^*‐HCOO* on In^3+^ and explains the lower intrinsic CH_3_OH formation rate.

This transient experiment also provided crucial insights into the origin of CO formation. During the CO_2_ phase, Cu/SiO_2_, Cu─In/SiO_2_ and Cu─Ga/SiO_2_ all produced both *κ^2^*‐HCOO* and CO* (on Cu^0^) as detectable surface species (Figures S18–S20). An analysis of their interplay revealed that the *κ^2^*‐HCOO* (Cu) signal decreased as the CO* signal increased. This provides strong evidence that *κ^2^*‐HCOO* (Cu) acts as an intermediate for CO* formation via decomposition (the RWGS pathway) as previously discussed. CO* was then rapidly converted upon the introduction of the H_2_ phase (the reason we could not observe it under steady state). In sharp contrast, CO* remained undetectable over Cu─Ga/SiO_2_ (Figure S20). This finding clearly demonstrates why Cu─Ga/SiO_2_ is so selective: the formate spillover and subsequent hydrogenation are so fast that this pathway completely outcompetes the parallel decomposition pathway on Cu, preventing CO formation.

While this study used a CO_2_/H_2_ feed, in technical CO/CO_2_/H_2_ syngas CO_2_ remains the immediate carbon source for methanol, with co‐fed CO acting mainly through the water‐gas shift to regenerate CO_2_ and scavenge product water [3]. The formate spillover pathway and the C─O/C─H bond‐strength descriptors established here are therefore expected to extend to CO‐blended feeds, with the CO/CO_2_ ratio additionally tuning the oxidation state of the dynamic Cu─M^+^ interface.

In summary, while Cu─Zn/SiO_2_ is the most active catalyst for methanol production, Cu─Ga/SiO_2_ achieves superior methanol selectivity by more effectively inhibiting the undesired CO side‐reaction via fast formate spillover. Conversely, Cu─In/SiO_2_ exhibits high selectivity but low activity due to the over‐stabilization of the formate after spillover, which inhibits further hydrogenation. As summarized schematically in Figure 8, these findings reveal how a promoter dictates product selectivity through control of formate spillover and stabilization. Ultimately, they provide a foundation for rational catalyst design: vibrational descriptors of the formate intermediate can guide the computational screening of promoters that balance intermediate stability against decomposition, while synthetic strategies that maximize the density of active interfacial sites can favor spillover over decomposition, together offering a route to further suppressing the unselective CO by‐product.

**FIGURE 8:**
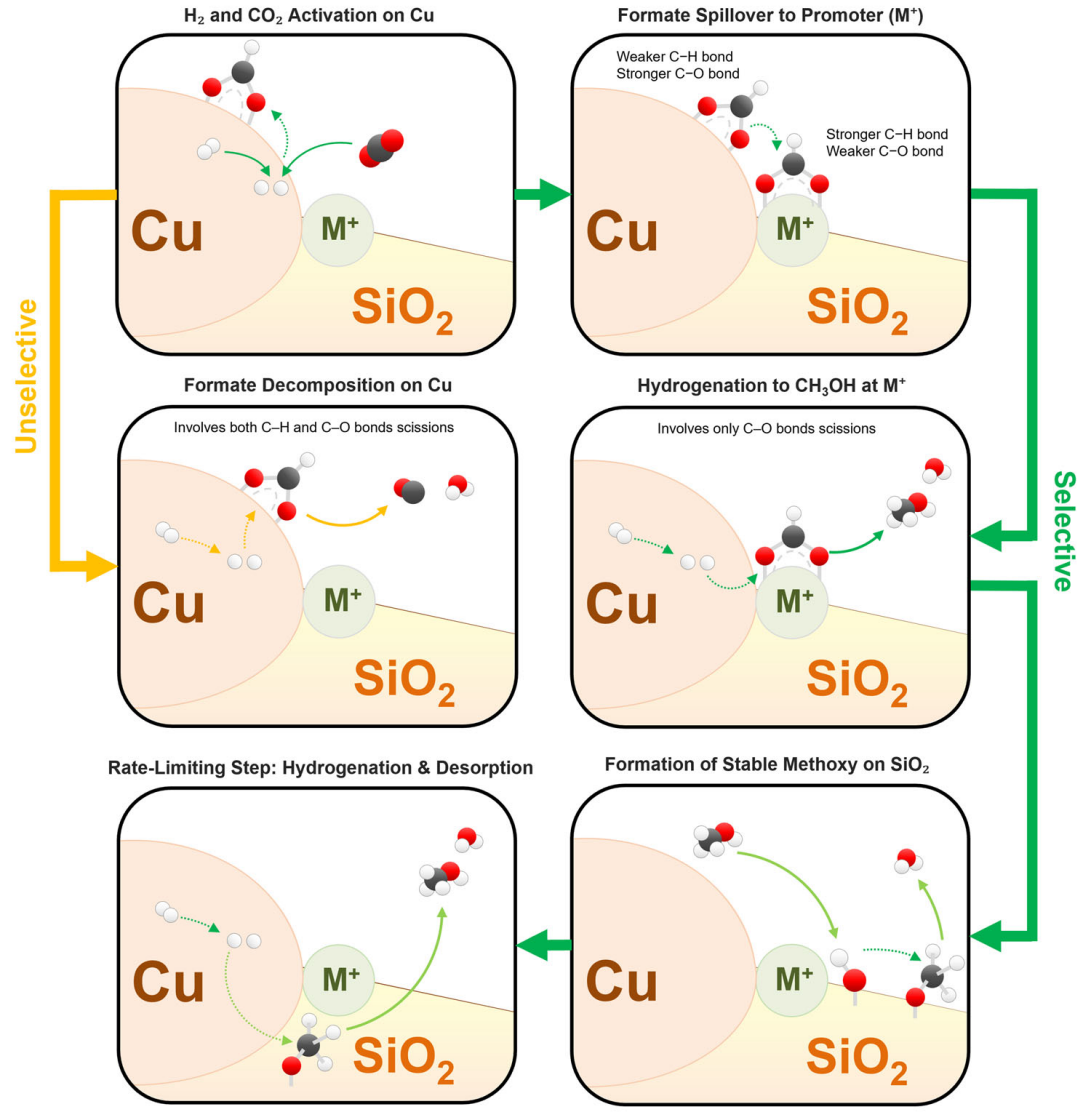
Proposed dual‐pathway mechanism for CO_2_ hydrogenation on Cu─M/SiO_2_ catalysts, detailing the molecular steps that control product selectivity. The unselective pathway (left) involves the decomposition of the formate intermediate on the Cu surface to produce CO. The selective pathway (right) is enabled by the crucial spillover of formate from Cu to an adjacent promoter site (M^+^). This protects the intermediate and initiates a reaction sequence that proceeds via a stable methoxy species on the SiO_2_ support, whose final hydrogenation to release methanol is the overall rate‐limiting step.

## 3 Conclusion

The Cu─M/SiO_2_ catalysts (M = Zn, Ga, and In) were prepared by surface organometallic chemistry to position isolated cationic M sites adjacent to Cu nanoparticles in the form of silicates. Although these promoters can initially form alloys upon reduction, in situ and ex situ XAS show they dynamically restructure into isolated interfacial cationic M sites under reaction conditions. *Operando* DRIFTS identified Cu‐bound formate as a common intermediate across all catalysts, with the cationic M sites enabling formate spillover from Cu to the promoter (Figure 8). Relative to unpromoted Cu/SiO_2_, where formate is prone to decomposition into CO, the cationic M sites stabilize the intermediate, suppressing decomposition and lowering CO formation.

This stabilization stems from the promoter's electronic effect on the adsorbed formate: it weakens the C─O bond and strengthens the C─H bond. Each bond strength then acts as a quantitative descriptor of a different performance metric: the C─O bond strength sets activity through a Sabatier‐type volcano, while the C─H bond strength varies linearly with methanol selectivity. Cu─Zn/SiO_2_ shows the highest activity, consistent with its optimal formate–Zn^2+^ binding on the Sabatier volcano, whereas the faster spillover to Ga^3+^ more effectively suppresses CO formation and gives the highest methanol selectivity. In contrast, In^3+^ binds formate too strongly after spillover, creating a kinetic trap that lowers activity.

All catalysts proceed through the same formate‐hydrogenation mechanism. Isotopic transient experiments showed negligible KIE for H_2_ dissociation, formate formation, and formate hydrogenation, but a significant KIE for methoxy hydrogenation/desorption to methanol, identifying this as the rate‐limiting step. These insights clarify the long‐debated Cu─ZnO synergy underlying industrial Cu/ZnO/Al_2_O_3_ catalysts and provide a foundation for rationally designing selective catalysts by tuning the interfacial environment and promoter acidity where formates form and are stabilized.

## Author Contributions

**Nat Phongprueksathat**: conceptualization, methodology, investigation, writing – original draft, data curation, software, formal analysis, validation, and visualization. **Gina Noh**: methodology, writing – review and editing, and investigation. **Scott R. Docherty**: investigation, writing – review and editing, and methodology. **Damian Vico van Berkel**: investigation, methodology, software, formal analysis, writing – review and editing, and validation. **Yannik Stiefel**: investigation, writing – review and editing, and methodology. **Christophe Copéret**: funding acquisition, writing – review and editing, project administration, resources, supervision, and methodology. **Atsushi Urakawa**: project administration, resources, supervision, methodology, conceptualization, funding acquisition, and writing – review and editing.

## Conflicts of Interest

The authors declare no conflicts of interest.

## Supporting information




**Supporting File**: Experimental procedures, commercial chemicals, catalyst preparation procedures, instrument specifications, and characterization techniques are covered in greater detail in the Supporting Information. The authors have also cited additional references within the Supporting Information [14, 15, 16, 17, 19, 23, 24, 39, 40, 41, 42, 43, 44, 45, 46, 47, 48, 49].

## Data Availability

The data that support the findings of this study are available on request from the corresponding author. The data are not publicly available due to privacy or ethical restrictions.
