# Supplementary material for: Key Drivers of Activity and Selectivity in Cu‐Based Catalysts for Methanol Synthesis From CO2: Insights From Atomically Dispersed Promoters
Source: Angew Chem Int Ed Engl. 2026 Aug 5;65(40):e2693293. doi: 10.1002/anie.2693293 (PMC13618279; doi:10.1002/anie.2693293)
Supplement: Supplementary file 1 [file ANIE-65-e2693293-s001.docx]

Supporting information

**Key Drivers of Activity and Selectivity in Cu-based Catalysts for Methanol Synthesis from CO_2_: Insights from Atomically Dispersed Promoters**

Nat Phongprueksathat,^[a,b]^ Gina Noh,^[c,d]^ Scott R. Docherty,^[c,e]^ Damian Vico van Berkel,^[a]^ Yannik Stiefel,^[c]^ Christophe Copéret,^[c]^ and Atsushi Urakawa*^[a]^

[a]	Dr. N. Phongprueksathat, D. Vico van Berkel, Prof. Dr. A. Urakawa
Catalysis Engineering
Department of Chemical Engineering
Delft University of Technology
Van der Maasweg 9, 2629 HZ Delft, The Netherlands
E-mail: [A.Urakawa@tudelft.nl](mailto:A.Urakawa@tudelft.nl)

[b]	Present Address: Dr. N. Phongprueksathat

Catalysis Engineering Group,

Van 't Hoff Institute for Molecular Sciences,

University of Amsterdam

Science Park 904, 1090 GD Amsterdam, The Netherlands

[c]	Dr. S.R. Docherty, Dr. G. Noh, Y. Stiefel, Prof. Dr. C. Copéret
Department of Chemistry and Applied Biosciences
ETH Zürich
Vladimir Prelog Weg 1-5, CH-8093 Zurich, Switzerland

[d]	Present Address: Dr. G. Noh
Department of Chemical Engineering
Pennsylvania State University
State College, PA, USA

[e]	Present Address: Dr. S.R. Docherty

Material Science

Department of Chemistry

Columbia University

3000 Broadway, Havemeyer Hall · New York, NY 10027

**Contents**

[**1.**	**Supplementary experimental**	3](#_Toc230753449)

[1.1	Materials and General Procedures	3](#_Toc230753450)

[1.2	Synthesis of Cu-In/SiO_2_ Catalyst	4](#_Toc230753451)

[1.2.1	Preparation of Indium-Decorated Silica (In/SiO_2_) Support	4](#_Toc230753452)

[1.2.2	Formation of Cu Nanoparticles on In/SiO_2_	4](#_Toc230753453)

[1.3	Catalyst Characterization Procedures	5](#_Toc230753454)

[**1.3.1**	**Elemental Analysis**	5](#_Toc230753455)

[**1.3.2**	**Fourier-Transform Infrared (FTIR) Spectroscopy**	5](#_Toc230753456)

[**1.3.3**	**X-ray Absorption Spectroscopy (XAS)**	5](#_Toc230753457)

[**1.3.4**	**Solid-State Nuclear Magnetic Resonance (ssNMR) Spectroscopy**	6](#_Toc230753458)

[1.4	Catalytic Testing Procedures	7](#_Toc230753459)

[1.5	*Operando* Diffuse Reflectance Infrared Fourier Transform Spectroscopy (DRIFTS) Experimental Procedures	8](#_Toc230753460)

[1.5.1	Temporal Evolution of Surface Species after Introducing H_2_ + CO_2_	9](#_Toc230753461)

[1.5.2	Steady-State Isotopic Transient Kinetic Analysis (SSITKA) Using D_2_ + CO_2_	9](#_Toc230753462)

[1.5.3	Transient Experiment Using He + CO_2_ (3:1) vs H_2_ + He (3:1)	10](#_Toc230753463)

[1.5.4	The Identification and Assignment of Each IR Band	11](#_Toc230753464)

[1.5.5	Harmonic Oscillator Model: Calculation of C–H/C–D Vibrational Frequency Ratio	12](#_Toc230753465)

[1.5.6	Multivariate Spectral Analysis	13](#_Toc230753466)

[1.5.7	Kinetic Isotope Effect	13](#_Toc230753467)

[1.5.8	Quantification of isotopic scrambling and surface–gas kinetic coupling	13](#_Toc230753468)

[**2.**	**Supplementary results**	15](#_Toc230753469)

[2.1	Catalyst Activity Testing Results	15](#_Toc230753470)

[2.2	Catalyst Characterization Results	16](#_Toc230753471)

[2.2.1	Elemental Analysis	16](#_Toc230753472)

[2.2.2	Fourier-Transform Infrared (FTIR) Spectroscopy	17](#_Toc230753473)

[2.2.3	X-ray Absorption Spectroscopy (XAS)	19](#_Toc230753474)

[2.2.4	Solid-State Nuclear Magnetic Resonance (ssNMR)	23](#_Toc230753475)

[2.3	*Operando* Diffuse Reflectance Infrared Fourier Transform Spectroscopy (DRIFTS) Results	24](#_Toc230753476)

[2.3.1	Evolution of Surface Species after Introducing H_2_ + CO_2_	24](#_Toc230753477)

[2.3.2	Mechanistic insights from the isotopic transient experiment	25](#_Toc230753478)

[2.3.3	Quantification of isotopic scrambling and surface–gas kinetic coupling	26](#_Toc230753480)

[2.3.4	Steady-State Isotopic Transient Kinetic Analysis (SSITKA) Using D_2_ + CO_2_	29](#_Toc230753481)

[2.3.5	Transient Experiment Using He + CO_2_ (3:1) vs H_2_ + He (3:1): Insights into Species Spillover	32](#_Toc230753482)

[References	35](#_Toc230753483)

1. **Supplementary experimental**
   1.1 Materials and General Procedures

All manipulations involving air- and moisture-sensitive compounds were performed under an inert argon atmosphere using standard Schlenk techniques or within an MBraun glovebox. Solvents such as pentane and toluene were dried using a two-column solvent purification system (MBraun MB-SPS-800, alumina columns), stored over activated 4Å molecular sieves, and degassed prior to use. Tetrahydrofuran (THF) and deuterated benzene (C_6_D_6_) were distilled from Na°/benzophenone under argon or vacuum, respectively, and stored over activated 4Å molecular sieves. Fumed SiO_2_ (Evonik AEROSIL 200) was typically pre-treated by calcination at 700 °C (973 K) under flowing synthetic air, followed by cooling under high vacuum (ca. 10^−3^ Pa) to yield dehydroxylated silica, denoted SiO_2-700_. The number of accessible hydroxyl groups was determined by titration methods.

Unless otherwise specified all preparations of catalysts were carried out under argon using standard Schlenk techniques and gloveboxes. Cu/SiO_2_,^[1]^ Cu-Zr/SiO_2_,^[1]^ Cu-Ga/SiO_2_,^[2]^ and Cu-Zn/SiO_2_^[3]^ were synthesized according to literature procedures and stored in an argon filled glovebox.

Similarly, the Cu-In/SiO_2_ was developed via In(OSi(O*t*Bu)_3_)_3_ from K. Samedov, *et al*.^[4]^ The indium precursor, indium tris(tris(tert-butoxy)siloxide) (In[OSi(OtBu)_3_]_3_), was synthesized following an adapted literature procedure involving the reaction of indium tris(hexamethyldisilazanide) with three equivalents of Tris(tert-butoxy)silanol ((tBuO)_3_SiOH) in refluxing toluene.

1.2 Synthesis of Cu-In/SiO_2_ Catalyst

The Cu-In/SiO_2_ catalyst was prepared using a sequential Surface Organometallic Chemistry (SOMC) approach in two main steps: preparation of the indium-decorated silica support, followed by the formation of copper nanoparticles.

1.2.1 Preparation of Indium-Decorated Silica (In/SiO_2_) Support

A solution of the indium precursor, In[OSi(OtBu)_3_]_3_ (synthesized as described in 1.1), in toluene was added to a slurry of SiO_2-700_ in toluene. The amount of precursor was calculated to achieve the target indium loading (e.g., ca. 0.1 mmol g^−1^ based on related M/SiO_2_ preparations). The suspension was typically stirred for several hours (ca. 12 h) at room temperature. After settling, the supernatant was decanted, and the resulting solid was washed multiple times with portions of toluene (3 x 10 mL) to remove unreacted precursor and byproducts. The material was then dried under vacuum (ca. 10^−3^ Pa) at room temperature.

To remove the residual organic ligands and generate the active indium sites, the grafted material was subjected to thermal treatment. The solid was heated under high vacuum (ca. 10^−3^ Pa) to 300 °C (ramp rate 5 °C min^−1^) and held for several hours (ca. 5 h). This process yielded the indium-decorated support, denoted In/SiO_2_, which contained isolated indium sites and regenerated surface silanol groups. The success of grafting and ligand removal was monitored by FTIR spectroscopy. The final indium loading was confirmed by ICP-OES.

1.2.2 Formation of Cu Nanoparticles on In/SiO_2_

Copper nanoparticles were subsequently generated on the prepared In/SiO_2_ support. A solution of the copper precursor, [Cu_n_(mesityl)_n_], in toluene was added to a slurry of the In/SiO_2_ support in the same solvent. The amount of copper precursor was calculated based on the target Cu loading and M/Cu ratio (e.g., M/Cu ≈ 0.264). The suspension was stirred for 12 h at room temperature. After stirring, the solvent was decanted, and the solid was washed thoroughly with pentane (3 x 10 mL). The resulting material was dried under vacuum (ca. 10^−3^ Pa) at room temperature.

The dried solid containing the grafted copper precursor was then reduced under flowing H_2_ (ca. 101 kPa, PanGas 99.999%). The material was heated to 300 °C (ramp rate 5 °C min^−1^) and held for 5 h. After reduction, the sample was cooled to room temperature under H_2_ flow, followed by evacuation under high vacuum (ca. 10^−3^ Pa). The final Cu-In/SiO_2_ catalyst was stored under an inert argon atmosphere in a glovebox. The formation of nanoparticles and removal of ligands were confirmed by FTIR. Copper loading was confirmed by ICP-OES.

1.3 Catalyst Characterization Procedures
    1.3.1 **Elemental Analysis**

Metal loadings (Cu and In content, wt%) were determined by inductively coupled plasma optical emission spectroscopy (ICP-OES), performed by Mikroanalytisches Labor Pascher, Remagen, Germany.

1.3.2 **Fourier-Transform Infrared (FTIR) Spectroscopy**

FTIR spectra of catalyst precursors and final materials were recorded on self-supporting wafers under an inert atmosphere using a Bruker spectrometer (Nicolet NEXUS 6700) in transmission mode (typically 4 cm^−1^ resolution, averaged scans). Spectra were typically normalized using the Si−O−Si overtone bands of the silica support. This technique was used to monitor the consumption of surface Si−OH groups and the appearance/disappearance of organic ligand C−H stretching and bending modes during grafting and thermal treatment steps.

1.3.3 **X-ray Absorption Spectroscopy (XAS)**

*Ex situ* and *in situ* XAS experiments were performed at the Cu K-edge and In K-edge to determine the oxidation state and coordination environment of the metals. Measurements were conducted at a synchrotron facility (SuperXAS beamline at the Swiss Light Source (SLS)).

*Ex situ* Samples: Powders were pressed into pellets of optimal thickness inside an argon-filled glovebox and sealed in airtight, X-ray transparent bags (e.g., aluminized plastic) to prevent air exposure before measurement. Reference materials (e.g., Cu foil, In foil, In_2_O_3_, relevant precursors) were measured under similar conditions.

*In situ* Samples: Catalyst powders (ca. 10-20 mg) were packed into a quartz capillary reactor cell connected to a gas flow system. The procedures are shown in the scheme below The catalyst was typically pre-treated *in situ* (e.g., reduction under 20% H_2_/Inert flow at 300-500 °C) before exposing it to reaction conditions (e.g., CO_2_/H_2_/Inert mixture at 230 °C, 5 bar). Spectra were collected periodically under reaction conditions to monitor changes.

**Figure S1** Experimental procedure for in situ XAS. Reduction condition: 300 °under 10 NmL min^−1^ of 20% H_2_ for 1 h. Reaction conditions: ca. 10 mg catalyst, H_2_/CO_2_ = 3, T = 230 °C, P = 5 bar, F_total_ = 10 NmL min^−1^.

**Data Analysis:** Spectra were background-corrected, normalized, and analyzed using standard XAS software packages (e.g., Demeter). XANES (X-ray Absorption Near Edge Structure) analysis focused on edge energy shifts to determine oxidation states.

1.3.4 **Solid-State Nuclear Magnetic Resonance (ssNMR) Spectroscopy**

Solid-state ^1^H and ^13^C NMR experiments were performed to identify surface species after reaction. Catalysts were typically reacted *ex situ* in a batch reactor under relevant conditions (e.g., 5 bar ^13^CO_2_/H_2_ 3:1 ratio, 230 °C, 12 h), cooled, evacuated, and packed into NMR rotors (e.g., 4 mm) inside an argon-filled glovebox. Spectra were recorded on a Bruker spectrometer (e.g., 400 MHz AVANCE III HD) using Magic Angle Spinning (MAS) (ca. 10 kHz). Cross-polarization (CP-MAS) techniques (^1^H-^13^C) with appropriate contact times (ca. 2 ms) and decoupling sequences (e.g., DUMBO for ^1^H-^13^C HETCOR) were employed. Chemical shifts were referenced using an external standard (e.g., adamantane).

1.4 Catalytic Testing Procedures

Catalytic CO_2_ hydrogenation reactions were performed in a fixed-bed tubular reactor (Stainless Steel 316, 9.1 mm inner diameter) operated in a down-flow configuration. Typically, 0.20-0.25 g of the catalyst powder (pre-oxidized in air) was diluted with 5.0 g of SiC (Fisher Scientific) and loaded into the reactor, supported by a sintered metal frit. For benchmark catalysts like Cu/ZnO/Al_2_O_3_, smaller amounts (20-30 mg) were used. The catalyst bed was positioned axially within the reactor. Temperatures were measured using a K-type thermocouple placed at the midpoint of the catalyst bed and controlled using a resistively heated furnace with electronic controllers. All gas transfer lines downstream of the reactor were heated (> 120 °C) to prevent product condensation. The reactor pressure was maintained using an electronic back-pressure regulator (Bronkhorst EL-PRESS). Gas flows (CO_2_, H_2_, and Ar inert) were controlled using electronic mass flow controllers (Bronkhorst EL-FLOW).

Prior to reaction, the catalysts were typically reduced *in situ*. A common procedure involved heating the catalyst under a flow of 15% H_2_ in N_2_ (50 NmL min^−1^) to 300 °C (ramp rate 5 °C min^−1^) and holding for 1-2 hours at atmospheric pressure. After reduction, the reactor was cooled to the reaction temperature (e.g., 230 °C) under an inert or H_2_ flow.

The reaction was initiated by introducing the feed gas mixture, typically consisting of CO_2_:H_2_:Ar in a 1:3:1 molar ratio , at the desired reaction pressure of 25 bar. Steady-state was often ensured by running under reaction conditions for a period (30 min to 3 hours) before collecting data.

Catalytic performance data were typically collected at differential CO_2_ conversions (e.g., <7%) to minimize heat and mass transfer limitations and product inhibition effects. Contact times were varied by adjusting the total gas flow rate (from 6 to 100 NmL min^−1^) while maintaining the constant feed composition and pressure. Catalyst stability was monitored by periodically returning to a reference flow rate condition throughout the experiment. Negligible deactivation was typically observed over extended periods (e.g., <5% rate decrease over 48 h).

The reactor effluent was analyzed using an online gas chromatograph (e.g., Agilent 7890N or 7890B). Product separation was achieved using packed columns (e.g., Restek Rt-U-BOND and Rt-Msieve 5A). Product concentrations were quantified using a flame ionization detector (FID) for organic compounds (CH_3_OH, Dimethyl Ether (DME)) and a thermal conductivity detector (TCD) for permanent gases (CO_2_, H_2_, Ar, CO, CH_4_). CH_4_ concentrations were typically below detection limits.

Reaction rates (r_Cu,x_) were calculated on a per-carbon basis and normalized by the mass of Cu in the catalyst bed. Product selectivity (S_x_) was defined on a per-carbon basis. CO_2_ conversion (X_CO2_) was calculated based on the moles of carbon in the products relative to the inlet moles of CO_2_. An inert gas (Ar) was used as an internal standard to correct for flow rate changes.

Since CH_3_OH formation rates often decreased with increasing residence time (indicating product inhibition), initial or intrinsic formation rates were determined by extrapolating the measured rates to zero residence time (or zero conversion). This was typically done using either an exponential fit to the shortest residence time data or a second/third-order polynomial fit to the rate vs. contact time data collected at low conversions (<7%). CO formation rates, being often less sensitive to residence time, were sometimes averaged across conditions to obtain initial rates.

1.5 *Operando* Diffuse Reflectance Infrared Fourier Transform Spectroscopy (DRIFTS) Experimental Procedures

The flow of gases (H_2_, CO_2_, and He) was controlled by 6 mass flow controllers (Bronkhorst). Switching between two reactant gas streams was done by a 4-way valve. The pressure of the two gas streams (to the cell and vent) was controlled by back pressure regulators (Bronkhorst). The outlet gas stream was analyzed by a Pfeiffer OmniStar GSD 300C mass spectrometer.

Before the measurements, the sample was reduced in situ at 300 °C in the H_2_ stream (20 NmL min^−1^ H_2_) for 1 h and subsequently cooled to a reaction temperature of 230 °C in the He stream. The cell was pressurized to 10-20 bar and immediately exposed to the reactant mixture (CO_2_:H_2_ = 1:3 molar ratio, total flow 20 NmL min^−1^) at the same pressure by the switching valve. The transient experiment utilizes a periodic perturbation of a system by external parameters (stimulation) to influence the concentration of active species.^[5]^ This experiment was performed in the above-mentioned setup by using a switching valve to change the stream of reactant gases to introduce the periodic concentration perturbation.

The catalyst powder (10-15 mg) was located in a cylindrical cavity (3 mm in diameter and 3 mm vertical length) of a custom-made high-pressure reaction cell (tested up to 40 bar). The cell was mounted in a Harrick Praying Mantis diffuse reflection (DRIFTS) accessory. The spectra were collected using a Thermo Scientific Nicolet 6700 FT-IR spectrometer equipped with a liquid-nitrogen-cooled MCT detector at 4 cm^−1^ resolution. The spectra were acquired continuously every 10 seconds in a time-resolved manner to monitor the reaction, stabilization process of the catalysts as well as evolution of surface species. No baseline correction was applied to the time-resolved spectra due to the baseline movement.

1.5.1 Temporal Evolution of Surface Species after Introducing H_2_ + CO_2_

**Figure S2** Experimental procedure for the temporal evolution of surface species Reduction condition: 300 °C under 10 NmL min^−1^ of H_2_ for 1 h. Reaction conditions: ca. 10 mg catalyst, H_2_/CO_2_ = 3, T = 230 °C, P = 10 bar, F_total_ = 10 NmL min^−1^.

1.5.2 Steady-State Isotopic Transient Kinetic Analysis (SSITKA) Using D_2_ + CO_2_

**Figure S3** Experimental procedure for SSITKA. Reduction condition: 300 °C under 10 NmL min^−1^ of H_2_ for 1 h. Reaction conditions: ca. 10 mg catalyst, H_2_/CO_2_ or D_2_/CO_2_ = 3, T = 230 °C, P = 10 bar, F_total_ = 10 NmL min^−1^.

1.5.3 Transient Experiment Using He + CO_2_ (3:1) vs H_2_ + He (3:1)

**Figure S4** Experimental procedures for the transient experiment. Reduction condition: 300 °C under 10 NmL min^−1^ of H_2_ for 1 h. Reaction conditions: ca. 10 mg catalyst, He/CO_2_ or H_2_/He = 3, T = 230 °C, P = 10 bar, F_total_ = 10 NmL min^−1^.

1.5.4 The Identification and Assignment of Each IR Band

**Table S1**. Assignment of surface species

| Wavenumber (cm^−1^) | Vibrational mode | Band assignment | Ref. |
| --- | --- | --- | --- |
| 2996 | *ν*_as_(C–H) | Adsorbed methanol (CH_3_OH*) | ^[6]^ |
| 2974 | *ν*(C–O) + *δ*(C–H) | Bidentate formate (*κ^2^*-HCOO*) on In_2_O_3_ | ^[7]^ |
| 2955-2961 | *ν*_as_(C–H) | Methoxy (CH_3_O*) on SiO_2_ | ^[6]^ |
| 2939 | *ν*_as_(C–H) | Methoxy (CH_3_O*) on In_2_O_3_ | ^[7]^ |
| 2939-2940 | *ν*_as_(C–H) | Methoxy (CH_3_O*) on ZnO | ^[6b]^ |
| 2937 | *ν*(C–O) + *δ*(C–H) | Bidentate formate (*κ^2^*-HCOO*) on Cu | ^[8]^ |
| 2923-2926 | *ν*_as_(C–H) | Methoxy (CH_3_O*) on Cu | ^[6a]^ |
| 2921 | *ν*_s_(C–H) | Adsorbed methanol (CH_3_OH*) | ^[6]^ |
| 2908-2917 | *ν*_as_(C–H) | Methoxy (CH_3_O*) on Ga_2_O_3_ | ^[9]^ |
| 2910-2915 | *ν*_as_(C–H) | Bridge formate (*µ*-HCOO*) on Ga_2_O_3_ | ^[9-10]^ |
| 2876-2897 | *ν*_s_(C–H) | Bridge formate (*µ*-HCOO*) on Ga_2_O_3_ | ^[9-10]^ |
| 2893 | *ν*(C–H) | Bidentate formate (*κ^2^*-HCOO*) on ZnO | ^[6b]^ |
| 2873 | *ν*(C–H) | Bidentate formate (*κ^2^*-HCOO*) on In_2_O_3_ | ^[7]^ |
| 2856-2857 | *ν*_s_(C–H) | Methoxy (CH_3_O*) on SiO_2_ | ^[6a]^ |
| 2855-2857 | *ν*(C–H) | Bidentate formate (*κ^2^*-HCOO*) on Cu | ^[8]^ |
| 2852 | *ν*_s_(C–H) | Adsorbed methanol (CH_3_OH*) | ^[6]^ |
| 2840 | *ν*_s_(C–H) | Methoxy (CH_3_O*) on In_2_O_3_ | ^[7]^ |
| 2829-2830 | *ν*_s_(C–H) | Methoxy (CH_3_O*) on ZnO | ^[6b]^ |
| 2815-2816 | *ν*_s_(C–H) | Methoxy (CH_3_O*) on Cu | ^[6a]^ |
| 2797 | *ν*_s_(C–H) | Methoxy (CH_3_O*) on Ga_2_O_3_ | ^[9]^ |
| 1600-1604 | *ν_as_*(C–O) | Bidentate formate (*κ^2^*-HCOO*) on Cu | ^[11]^ |
| 1585-1589 | *ν_as_*(C–O) | Bidentate formate (*κ^2^*-HCOO*) on ZnO | ^[11]^ |
| 1363–1377 | *ν_a_*(C–O) | Bidentate formate (κ^2^-HCOO*) on ZnO | ^[11]^ |
| 1352–1358 | *ν_a_*(C–O) | Bidentate formate (κ^2^-HCOO*) on Cu | ^[11]^ |
| 1080 | *ν*(C–O) | Methoxy (CH_3_O*) on ZnO | ^[6]^ |
|  |  |  |  |

1.5.5 Harmonic Oscillator Model: Calculation of C–H/C–D Vibrational Frequency Ratio

The approximate frequency shift for a C–D bond relative to a C–H bond can be calculated using the harmonic oscillator model. Assuming the force constant of the bond remains the same upon isotopic substitution, the vibrational frequency ($v$) is inversely proportional to the square root of the reduced mass ($\mu$).

The ratio of the C–D frequency to the C–H frequency is therefore given by the inverse ratio of the square root of their reduced masses:

$$v_{C-D}/v_{C-H}=\sqrt{\mu_{C-H}/\mu_{C-D}}$$

The reduced mass ($\mu$) for two atoms with masses $m_{1}$ and $m_{2}$ is calculated as:

$$\mu=\frac{m_{1}\times m_{2}}{m_{1}+m_{2}}$$

Using the approximate atomic masses for Carbon ($m_{C}\approx12.0\text{ amu}$), Hydrogen ($m_{H}\approx1.0\text{ amu}$), and Deuterium ($m_{D}\approx2.0\text{ amu}$), the reduced masses for the C–H and C–D bonds are calculated as follows:

Reduced mass for C–H bond: $\mu_{C-H}=\frac{12.0\times1.0}{12.0+1.0}=\frac{12.0}{13.0}\approx0.923\text{ amu}$

Reduced mass for C–D bond: $\mu_{C-D}=\frac{12.0\times2.0}{12.0+2.0}=\frac{24.0}{14.0}\approx1.714\text{ amu}$

Using these values, the expected frequency ratio is:

$v_{C-D}/v_{C-H}=\sqrt{\frac{0.923\text{ amu}}{1.714\text{ amu}}}\approx\sqrt{0.5385}\approx0.734$

This calculated factor of ~0.73 is the theoretical ratio by which the C–D stretching frequency is lower than the corresponding C–H stretching frequency. This provides a reliable approximation for assigning the isotopically shifted bands observed in the DRIFTS spectra.

1.5.6 Multivariate Spectral Analysis

Multivariate spectral analysis is performed by the Multivariate Curve Resolution-Alternating Least Squares (MCR-ALS) algorithm, as described elsewhere.^[12]^ MCR is a chemometric method used for better data processing and deconvolution of complex spectra down to individual components based on kinetic resolution. It can deliver the pure response profiles (e.g. spectra, pH profiles, time profiles, elution profiles) of the chemical species of an unresolved mixture when no previous information is available about the nature and composition of these mixtures.

1.5.7 Kinetic Isotope Effect

An estimation of the KIE value for the methoxy transients was obtained by linearization of the MCR-resolved concentration profiles and a subsequent extraction of the slope (k) within the linear regime, assuming a pseudo-zero-order rate constant:

$$k_{H/D}= \frac{d\theta_{{C(H/D)}_{3}O*}}{dt}=slope$$

The KIE hence follows:

$$KIE=\frac{k_{{CH}_{3}O*}}{k_{{CD}_{3}O*}}$$

1.5.8 Quantification of isotopic scrambling and surface–gas kinetic coupling

To quantify the extent and kinetics of isotopic scrambling during the SSITKA experiment, the transient responses of all four methanol isotopologues were analyzed at both isotopic switches. Switch times were programmed (5 min and 185 min) and synchronized to the DRIFTS acquisition; the delay between the programmed switch and the gas-phase response at the mass spectrometer reflects the residence-time distribution (RTD) of the cell and downstream lines. The RTD was estimated from the step responses of H_2_ and D_2_ themselves, which act as near-ideal inert tracers at the differential conversions employed (<5%), giving a mean mixing time
constant 𝜏_RTD_ = 4.9 min (averaged over four responses). Gas-phase time constants were extracted by fitting RTD-convolved exponential models; where the observed response was faster than the RTD (and deconvolution is therefore ill-conditioned).

$$\text{rise:}\quad\boldsymbol{y}\left( \boldsymbol{t} \right)\boldsymbol{=b+A}\left( \boldsymbol{1-}\boldsymbol{e}^{\boldsymbol{-}\left( \boldsymbol{t-}\boldsymbol{t}_{\boldsymbol{d}} \right)\boldsymbol{/}\boldsymbol{\tau}_{\text{RTD}}} \right)$$

$$\text{decay:}\quad\boldsymbol{y}\left( \boldsymbol{t} \right)\boldsymbol{=b+A}\boldsymbol{e}^{\boldsymbol{-}\left( \boldsymbol{t-}\boldsymbol{t}_{\boldsymbol{d}} \right)\boldsymbol{/}\boldsymbol{\tau}_{\text{RTD}}}\quad\text{for }\boldsymbol{t}\boldsymbol{\geq}\boldsymbol{t}_{\boldsymbol{d}}\boldsymbol{.}$$

2. **Supplementary results**
   2.1 Catalyst Activity Testing Results

**Table S2**. Complete raw data for CO_2_ conversion, CH_3_OH and CO selectivity used in **Figure 2a**

| **Catalyst** | **Data Point** | **CO_2_ Conversion (%)** | **CH_3_OH Selectivity (%)** | **CO Selectivity (%)** |
| --- | --- | --- | --- | --- |
| Cu-Ga/SiO_2_ | 1 | 0.61 | 92.42 | 7.58 |
|  | 2 | 0.82 | 92.86 | 7.14 |
|  | 3 | 0.98 | 92.36 | 7.64 |
|  | 4 | 1.27 | 92.23 | 7.77 |
|  | 5 | 1.70 | 91.85 | 8.15 |
|  | 6 | 2.08 | 91.28 | 8.72 |
|  | 7 | 2.32 | 91.06 | 8.94 |
|  | 8 | 2.75 | 90.19 | 9.81 |
|  | 9 | 2.90 | 89.88 | 10.12 |
|  | 10 | 3.14 | 89.23 | 10.77 |
| Cu-Zn/SiO_2_ | 1 | 1.39 | 80.26 | 19.74 |
|  | 2 | 1.50 | 81.61 | 18.39 |
|  | 3 | 1.71 | 81.10 | 18.90 |
|  | 4 | 2.11 | 79.75 | 20.25 |
|  | 5 | 2.80 | 77.79 | 22.21 |
|  | 6 | 3.36 | 76.06 | 23.94 |
|  | 7 | 3.79 | 75.54 | 24.46 |
|  | 8 | 4.42 | 73.44 | 26.56 |
|  | 9 | 4.74 | 72.99 | 27.01 |
|  | 10 | 5.19 | 71.28 | 28.72 |
| Cu/Zr/SiO_2_ | 1 | 1.00 | 69.20 | 30.80 |
|  | 2 | 1.02 | 69.92 | 30.08 |
|  | 3 | 1.26 | 66.89 | 33.11 |
|  | 4 | 1.67 | 63.71 | 36.29 |
|  | 5 | 2.45 | 58.17 | 41.83 |
|  | 6 | 3.30 | 52.72 | 47.28 |
|  | 7 | 3.99 | 48.85 | 51.15 |
|  | 8 | 5.23 | 42.74 | 57.26 |
|  | 9 | 6.02 | 39.14 | 60.86 |
|  | 10 | 7.10 | 34.94 | 65.06 |
| Cu/SiO_2_ | 1 | 0.37 | 46.60 | 53.40 |
|  | 2 | 0.37 | 48.39 | 51.61 |
|  | 3 | 0.49 | 47.29 | 52.71 |
|  | 4 | 0.76 | 47.15 | 52.85 |
|  | 5 | 1.28 | 46.50 | 53.50 |
|  | 6 | 1.81 | 44.63 | 55.37 |
|  | 7 | 2.41 | 42.65 | 57.35 |
|  | 8 | 3.38 | 38.21 | 61.79 |
|  | 9 | 4.00 | 36.18 | 63.82 |
|  | 10 | 4.89 | 32.65 | 67.35 |
| Cu-In/SiO_2_ | 1 | 0.05 | 80.09 | 19.91 |
|  | 2 | 0.05 | 74.65 | 25.35 |
|  | 3 | 0.07 | 74.68 | 25.32 |
|  | 4 | 0.11 | 75.00 | 25.00 |
|  | 5 | 0.17 | 75.97 | 24.03 |
|  | 6 | 0.25 | 76.78 | 23.22 |
|  | 7 | 0.32 | 78.02 | 21.98 |
|  | 8 | 0.45 | 79.35 | 20.65 |
|  | 9 | 0.54 | 80.83 | 19.17 |
|  | 10 | 0.68 | 82.28 | 17.72 |

Rates were measured for In/SiO_2_; product concentrations in the reactor effluent were below detection limits for all reaction conditions.

**Table S3.** Intrinsic formation rates and selectivity for CH_3_OH and CO used in **Figure 2b**.

| **Catalyst** | **CH_3_OH Formation Rate (µmol s^−1^ g_Cu_^−1^)** | **CO Formation Rate (µmol s^−1^ g_Cu_^−1^)** | **Intrinsic CH_3_OH Selectivity (%)** | **Intrinsic CO Selectivity (%)** |
| --- | --- | --- | --- | --- |
| Cu/SiO_2_ | 3.6 | 3.8 | 48.9 | 51.1 |
| Cu-In/SiO_2_ | 2.1 | 0.6 | 77.5 | 22.5 |
| Cu/Zr/SiO_2_ | 13.0 | 3.4 | 79.4 | 20.6 |
| Cu-Zn/SiO_2_ | 16.0 | 3.1 | 83.6 | 16.4 |
| Cu-Ga/SiO_2_ | 15.0 | 0.9 | 94.4 | 5.6 |

Pretreatment was performed at 300 °C under 20 NmL min^−1^ of H_2_ for 2 h.

Reaction conditions are ca. 10 mg catalyst, 230 °C, 25 bar, H_2_/CO_2_ = 3, total flow rate 6-100 NmL min^−1^, TOS = 180 min.

2.2 **Catalyst Characterization Results**
    2.2.1 Elemental Analysis

The final Cu and In loadings for the synthesized Cu-In/SiO_2_ catalyst were determined by ICP-OES and are reported as 3.81 wt% Cu and 1.82 wt% In, corresponding to an In/Cu atomic ratio of 0.264.

Table S4. Physicochemical properties of the Cu/SiO_2_ and Cu-M/SiO_2_ (M = Zr, Zn, Ga, In) catalysts prepared via SOMC.

| Material | Cu (wt%)ᵃ | M (wt%)^a^ | M/Cu (at.) | S_BET (m² g^−1^)^b^ | N_2_O (μmol g^−1^)^c^ | d_TEM (nm) | Reference |
| --- | --- | --- | --- | --- | --- | --- | --- |
| Cu/SiO_2_ | 4.19 | – | – | 197 | 50 | 2.9 ± 1.3 | ^[1]^ |
| Cu-Zr/SiO_2_ | 4.19 | 0.96 | 0.16 | 206 | 50 | 2.6 ± 1.3 | ^[1]^ |
| Cu-Zn/SiO_2_ | 4.16 | 1.62 | 0.40 | 187 | 52 | 3.9 ± 1.0 | ^[3]^ |
| Cu-Ga/SiO_2_ | 3.88 | 1.61 | 0.40 | 200 | 55 | 4.6 ± 1.4 | ^[2]^ |
| Cu-In/SiO_2_ | 3.81 | 1.82 | 0.26 | – | 57 | 2.6 ± 1.3 | This work |

^a^ICP-OES; ^b^N_2_ physisorption, BET; ^c^N_2_O titration on fresh catalyst (1:2 N_2_O:Cu_s_); ^d^TEM, log-normal fit;

2.2.2 Fourier-Transform Infrared (FTIR) Spectroscopy

FTIR spectra confirmed the successful grafting of the indium precursor onto SiO_2_-700 by the consumption of isolated silanol bands (ν(O−H) ~3747 cm^−1^) and the appearance of C−H stretching bands from the precursor ligands (Figure S5).

**Figure S5** IR spectra throughout the synthesis of In@SiO_2_. (Bottom) SiO_2_-700 support, partially dehydroxylated at 700 °C. (Middle) The "Grafted" material, after contacting SiO_2_-700 with a solution of In(OSi(OtBu)_3_)_3_ and drying. (Top) The final In@SiO_2_ material after thermal treatment under vacuum at 300 °C.

Subsequent thermal treatment under vacuum resulted in the removal of these C−H bands and the reappearance of Si−OH bands. Similarly, grafting of the copper precursor onto In/SiO_2_ resulted in further consumption of Si−OH bands and the appearance of C−H bands associated with the OtBu ligands, which were subsequently removed upon thermal treatment under H_2_, restoring the Si−OH features (Figure S6).

**Figure S6** IR spectra throughout the synthesis of Cu-In/SiO_2_. (Bottom) The In@SiO_2_ support. (Middle) The "Grafted" material, after contacting In@SiO_2_ with a solution of [Cuₓ(mes)ₓ]. (Top) The final Cu-In/SiO_2_ catalyst after thermal treatment under H_2_ at 300 °C.

2.2.3 X-ray Absorption Spectroscopy (XAS)

**Figure S7** EXAFS fits in R-space (Fourier-transform magnitude, not phase-corrected) of as-prepared Cu-In/SiO_2_ at **a** the Cu K-edge and **b** the In K-edge from a simultaneous two-edge fit (Cu–In and In–Cu paths constrained to share R and σ^2^), and **c** the In/SiO_2_ reference at the In K-edge.

**Table S5.** EXAFS fit parameters (coordination number N, interatomic distance R, and Debye–Waller factor σ^2^) for reduced Cu-In/SiO_2_ from the Cu K-edge fit and the simultaneous Cu/In K-edge fit, and for the In/SiO_2_ reference material at the In K-edge.

Cu K-edge fit of Cu-In/SiO_2_

| **Path** | **N** | **R (Å)** | **σ^2^ (Å^2^)** |
| --- | --- | --- | --- |
| Cu–Cu | 6.4 ± 0.7 | 2.48 ± 0.01 | 0.013 |
| Cu–In | 2.1 ± 1.8 | 2.71 ± 0.02 | 0.025 |

The simultaneous Cu and In K-edge fit of Cu-In/SiO_2_

| **Path** | **N** | **R (Å)** | **σ^2^ (Å^2^)** |
| --- | --- | --- | --- |
| Cu–Cu | 6.3 ± 0.5 | 2.475 ± 0.007 | 0.013 |
| Cu–In | 1.7 ± 0.6 | 2.69 ± 0.01* | 0.020* |
| In–O | 5.3 ± 1.6 | 2.16 ± 0.02 | 0.030 |
| In–Cu | 2.1 ± 0.7 | 2.69 ± 0.01* | 0.020* |

(* = shared between Cu and In edges)

In K-edge fit of the reference material In/SiO_2_.

| **Path** | **N** | **R (Å)** | **σ^2^ (Å^2^)** |
| --- | --- | --- | --- |
| In–O (short) | 3 | 2.05 ± 0.01 | 0.004 |
| In–O (long) | 2 | 2.53 ± 0.02 | 0.008 |
| In–Si | 2 | 2.93 ± 0.02 | 0.005 |

**Figure S8** *Ex situ* In K-edge XANES spectra recorded after air exposure (purple), after reduction with H_2_ (green), and after reaction with CO_2_ + H_2_ (yellow). The spectrum of In_2_O_3_ is included as a reference (dashed grey). Pretreatment was performed at 300 °C under 10 NmL min^−1^ of 20% H_2_ for 1 h. Reaction conditions are ca. 10 mg catalyst, 230 °C, 5 bar, H_2_/CO_2_ = 3, total flow rate 20 NmL min^−1^, TOS = 60 min.

**Table S6.** Quantitative comparison of promoter speciation in Cu-M/SiO_2_ catalysts (M = Zn, Ga, In) prepared via SOMC, after H_2_ reduction and under CO_2_ hydrogenation conditions. Method, fitted/reference values, fraction of M in the CuM_x_ alloy phase (*f*_alloy_), and source.

| **Catalyst** | **Method** | **After H_2_ reduction** | | **Under reaction (5 bar, 230 °C, CO_2_:H_2_:N_2_ = 1:3:1)** | | **Source** |
| --- | --- | --- | --- | --- | --- | --- |
|  |  | Measured value | *f*_alloy_ | Measured value | *f*_alloy_ |  |
| **Cu-Zn/SiO_2_** | Linear-combination XANES fit at Zn K-edge (references: Zn^2+^/SiO_2_, α-brass) | 51% Zn^2+^ + 49% α-brass^a^ | **~49%** | 84% Zn^2+^ + 16% α-brass | **~16%** (partial de-alloying) | ^[3]^ SI Figs. S8, S21, S22 |
| **Cu-Ga/SiO_2_** | EXAFS coordination at Ga K-edge N(Ga–O) in Cu-Ga/SiO_2_ vs. parent Ga^3+^/SiO_2_ | N(Ga–O) = 2 ± 1 vs. 4 in Ga/SiO_2_ | **~50%** CuGa_x_, x ≈ 0.2 | Reduced-Ga XANES feature absent; white line fully restored | **~0%** (complete de-alloying) | ^[2]^ Table 1, Fig. 4 |
| **Cu-In/SiO_2_** | Simultaneous EXAFS fit at Cu and In K-edges Cu–In and In–Cu paths share R and σ^2^ | N(In–Cu) = 2.1 ± 0.7 at R = 2.69 Å^b^ | **~25–40%**^c^ CuIn_x_, low x | In K-edge edge energy = 27940.5 eV (≈ In_2_O_3_) | **~0%** (essentially complete de-alloying) | **this work** Figs. S7, S8; Table S5 |
| **Cu-Zr/SiO_2_** (reference) | EXAFS at Zr K-edge isolated Zr^4+^ sites, non-reducible promoter | Zr^4+^ only no alloy formation | **0%** | Zr^4+^ only | **0%** (static interface) | ^[1]^ |

^a^ After the in-reactor pretreatment protocol (exposure to air followed by re-reduction at 300 °C under H_2_), the alloyed fraction in Cu-Zn/SiO_2_ decreases from 49% (as-prepared at 500 °C) to 29% (linear-combination fit using Zn^2+^/SiO_2_ and α-brass; ref [3], SI Fig. S21).

^b^ The Cu–In/In–Cu distance of 2.69 Å is shorter than in any stoichiometric Cu–In intermetallic phase (2.75 Å in Cu_2_In, 2.83 Å in CuIn), indicating that In is dispersed as a dilute substitutional dopant within Cu nanoparticles (CuIn_x_ solid solution with low x) rather than forming a discrete intermetallic compound. The corresponding contraction of the Cu–Cu distance to 2.475 Å (vs. 2.55 Å in fcc Cu) is a structural fingerprint of this substitutional doping.

^c^ Alloyed fraction estimated from the In K-edge coordination number N(In–Cu) = 2.1 ± 0.7 by assuming two populations: alloyed In (with N_full_ Cu neighbors) and Inᴵᴵᴵ on silica (with 5 oxygen and 0 Cu neighbors, from the parent In/SiO_2_ reference). The bounds reflect a physically reasonable range of N_full_ = 6–8, consistent with the average Cu coordination of 8.0 (= 6.3 + 1.7) obtained for the Cu nanoparticles in the same fit. A tighter bound on *f*_alloy_ would require a linear-combination fit using an In foil reference (planned follow-up).

2.2.4 Solid-State Nuclear Magnetic Resonance (ssNMR)

The spectra are dominated by signals for methoxy (CH_3_O*) species (δ ≈ 49-50 ppm). A small signal for dimethylether (DME) is also observed for Cu-Ga/SiO_2_. Notably, no signals for stable formate species (expected at δ ≈ 170 ppm) are detected for any of the three catalysts.

**Figure S9** Solid-state CP-MAS ^13^C NMR spectra (400 MHz, 100.6 MHz for ^13^C) of Cu-Zn/SiO_2_ (blue), Cu-Ga/SiO_2_ (red), and Cu-In/SiO_2_ (green) catalysts. The samples were reacted *ex situ* with a ^13^CO_2_/H_2_ mixture (1:3 ratio, 5 bar) at 230 °C for 12 h prior to analysis. All spectra were recorded using a 4.0 mm rotor at a spinning rate of 10 kHz.

2.3 *Operando* Diffuse Reflectance Infrared Fourier Transform Spectroscopy (DRIFTS) Results
    2.3.1 Evolution of Surface Species after Introducing H_2_ + CO_2_

**Figure S10** Time-resolved *operando* DRIFT spectra of the surface species formed during the CO_2_ hydrogenation reaction over Cu/SiO_2_, Cu-Zn/SiO_2_, Cu-Ga/SiO_2_, Cu-In/SiO_2_ catalysts. Pretreatment was performed at 300 °C under 10 NmL min^−1^ of H_2_ for 2 h. Reaction conditions are ca. 10 mg catalyst, 230 °C, 10 bar, H_2_/CO_2_ = 3, total flow rate 10 NmL min^−1^.

2.3.2 Mechanistic insights from the isotopic transient experiment

**Figure S11** Transient responses of surface species and gas products during the steady-state isotopic switching from CO_2_ + H_2_ to CO_2_ + D_2_ or from CO_2_ + D_2_ to CO_2_ + H_2_: Corresponding normalized ion current signal of isotope-labeled products. Reaction conditions: 10 mg catalyst, H_2_(or D_2_)/CO_2_ = 3, T = 230 °C, P = 10 bar, F_total_ = 10 NmL min^−1^.

**Figure S12** Linearized decaying methoxy surface responses used for the extraction of the rate constants for a) CH_3_O* and b) CD_3_O*. Reaction conditions: 10 mg catalyst, H_2_/CO_2_ = 3, T = 230 °C, P = 10 bar, F_total_ = 10 NmL min^−1^.

2.3.3 Quantification of isotopic scrambling and surface–gas kinetic coupling

Two regimes are observed. The primary products (CD_3_OD under CO_2_ + D_2_; CH_3_OH under CO_2_ + H_2_) rise monotonically with time constants of 24 min and 7 min, respectively. The scrambled isotopologues CH_3_OD and CD_3_OH instead display a peak-and-decay response: they rise sharply within the first few minutes after the switch and then decay toward a low steady-state plateau. This transient behavior is consistent with the consumption of a finite, slowly-replenished surface H (or D) reservoir. The decay time constant of the scrambled species (𝜏_d_ ≈ 23 min at the H_2_→D_2_ switch) is comparable to the rise time constant of the primary product (≈ 24 min), indicating that both populations are released through the common rate-limiting methoxy hydrogenation/desorption step. We note that the relative intensities and rise profiles of CH_3_OD and CD_3_OH are additionally complicated by the inability of MS to distinguish CH_3_OD from CH_2_DOH at m/z = 33 (see below) and by the different number of new C–H/C–D bonds each isotopologue requires; we therefore do not interpret the individual scrambled-species rise rates mechanistically, and base the conclusion that carbon hydrogenation is not rate-limiting on the near-simultaneous appearance of CD_3_OH and CH_3_OD reported in the main text (**Figure S11a**).

At steady state, the primary isotopologue dominates the methanol signal: 86% CD_3_OD under CO_2_ + D_2_ and 96% CH_3_OH under CO_2_ + H_2_, with scrambled isotopologues accounting for 14% and 4%, respectively (**Figure S13e**). Because the m/z = 33 channel cannot distinguish CH_3_OD from CH_2_DOH, its intensity is taken as a conservative upper bound on partial-scrambling products; even so, the fully-labeled primary product dominates and the mechanistic conclusions are unaffected.

The MCR-resolved surface species (**Figure S14**) corroborate this picture. Surface formate (*κ^2^*-(H/D)COO* on Cu and Zn^2^⁺) exchanges rapidly upon each switch (𝜏 < 1 min, at the limit of the spectral acquisition rate), confirming that formate formation and exchange are not rate-limiting. Surface methoxy exchanges far more slowly and is best described by two kinetically distinct populations: a reactive pool that exchanges on the minute timescale and a slowly-exchanging pool (𝜏 ≈ 1 h) that does not reach equilibrium within the 180 min observation window and is tentatively assigned to spectator methoxy on remote silica sites. The productive, rate-limiting hydrogenation/desorption is associated with the reactive pool, whose exchange timescale (convolved with the RTD) is consistent with the observed gas-phase methanol rise.

**Figure S13** Transient responses of the four methanol isotopologues during steady-state isotopic switching over Cu-Zn/SiO_2_, with RTD-convolved exponential fits. **a**, **c** Primary products and **b**, **d** scrambled isotopologues at the H_2_ + CO_2_ → D_2_ + CO_2_ switch (**a**, **b**) and the D_2_ + CO_2_ → H_2_ + CO_2_ switch (**c**, **d**). Dashed lines mark the programmed switch times. Time constants marked with an asterisk are residence-time-limited and are reported as observed values. **e** Background-subtracted steady-state methanol composition under each feed. Reaction conditions: 10 mg catalyst, H_2_/CO_2_ = 3, T = 230 °C, P = 10 bar, F_total_ = 10 NmL min^−1^.

**Figure S14** Kinetics of MCR-resolved surface species during isotopic switching over Cu-Zn/SiO_2_. **a**, **b** Surface formate (*κ^2^*-HCOO*/*κ^2^*-DCOO* on Cu-Zn/SiO_2_) exchanges rapidly (𝜏 < 1 min) at both switches. **c**, **d** Surface methoxy (CH_3_O*/CD_3_O* on SiO_2_) exchanges slowly and is described by a bi-exponential model with a fast (reactive) and a slow (spectator) pool. **e** Comparison of all extracted time constants on a logarithmic axis; the shaded band marks the residence-time-limited region for the gas-phase measurement. Reaction conditions: 10 mg catalyst, H_2_(or D_2_)/CO_2_ = 3, T = 230 °C, P = 10 bar, F_total_ = 10 NmL min^−1^.

2.3.4 Steady-State Isotopic Transient Kinetic Analysis (SSITKA) Using D_2_ + CO_2_

**Figure S15** Transient responses of surface species and gas products during transient isotopic switching from CO_2_ + D_2_ to CO_2_ + H_2_ over Cu/SiO_2_. Time-resolved DRIFT spectra of ν(C–H) region and ν(C–D) region. Component spectra obtained by MCR were applied to the time-resolved DRIFT spectra. Concentration profiles of the spectra of the corresponding components obtained by MCR. Corresponding normalized ion current signal of isotope-labeled products. Reaction conditions: 10 mg catalyst, H_2_(or D_2_)/CO_2_ = 3, T = 230 °C, P = 10 bar, F_total_ = 10 NmL min^−1^.

**Figure S16** Transient responses of surface species and gas products during transient isotopic switching from CO_2_ + D_2_ to CO_2_ + H_2_ over Cu-Ga/SiO_2_. Time-resolved DRIFT spectra of ν(C–H) region and ν(C–D) region. Component spectra obtained by MCR were applied to the time-resolved DRIFT spectra. Concentration profiles of the spectra of the corresponding components obtained by MCR. Corresponding normalized ion current signal of isotope-labeled products. Reaction conditions: 10 mg catalyst, H_2_(or D_2_)/CO_2_ = 3, T = 230 °C, P = 10 bar, F_total_ = 10 NmL min^−1^.

**Figure S17** Transient responses of surface species and gas products during transient isotopic switching from CO_2_ + D_2_ to CO_2_ + H_2_ over Cu-In/SiO_2_. Time-resolved DRIFT spectra of ν(C–H) region and ν(C–D) region. Component spectra obtained by MCR were applied to the time-resolved DRIFT spectra. Concentration profiles of the spectra of the corresponding components obtained by MCR. Corresponding normalized ion current signal of isotope-labeled products. Reaction conditions: 10 mg catalyst, H_2_(or D_2_)/CO_2_ = 3, T = 230 °C, P = 10 bar, F_total_ = 10 NmL min^−1^.

2.3.5 Transient Experiment Using He + CO_2_ (3:1) vs H_2_ + He (3:1): Insights into Species Spillover

**Figure S18** Transient responses of surface species and gas products during switching from He + CO_2_ to H_2_ + He over Cu/SiO_2_ catalysts: Component spectra obtained by MCR applied on the time-resolved DRIFT spectra. Concentration profiles of the spectra of the corresponding components obtained by MCR. Corresponding normalized ion current signal of products. Reaction conditions: 10 mg catalyst, H_2_/CO_2_ = 3, T = 230 °C, P = 20 bar, F_total_ = 10 NmL min^−1^.

**Figure S19** Transient responses of surface species and gas products during switching from He + CO_2_ to H_2_ + He over Cu-In/SiO_2_ catalysts: Component spectra obtained by MCR applied on the time-resolved DRIFT spectra. Concentration profiles of the spectra of the corresponding components obtained by MCR. Corresponding normalized ion current signal of products. Reaction conditions: 10 mg catalyst, H_2_/CO_2_ = 3, T = 230 °C, P = 20 bar, F_total_ = 10 NmL min^−1^.

**Figure S20** Transient responses of surface species and gas products during switching from He + CO_2_ to H_2_ + He over Cu-Ga/SiO_2_ catalysts: Component spectra obtained by MCR applied on the time-resolved DRIFT spectra. Concentration profiles of the spectra of the corresponding components obtained by MCR. Corresponding normalized ion current signal of products. Reaction conditions: 10 mg catalyst, H_2_/CO_2_ = 3, T = 230 °C, P = 20 bar, F_total_ = 10 NmL min^−1^.

References

[1]	E. Lam, K. Larmier, P. Wolf, S. Tada, O. V. Safonova, C. Copéret, *Journal of the American Chemical Society* **2018**, *140*, 10530–10535.

[2]	E. Lam, G. Noh, K. W. Chan, K. Larmier, D. Lebedev, K. Searles, P. Wolf, O. V. Safonova, C. Copéret, *Chemical Science* **2020**, *11*, 7593–7598.

[3]	E. Lam, G. Noh, K. Larmier, O. V. Safonova, C. Copéret, *Journal of Catalysis* **2021**, *394*, 266–272.

[4]	K. Samedov, Y. Aksu, M. Driess, *ChemPlusChem* **2012**, *77*, 663–674.

[5]	A. Urakawa, T. Bürgi, A. Baiker, *Chemical Engineering Science* **2008**, *63*, 4902–4909.

[6]	aG. J. Millar, C. H. Rochester, K. C. Waugh, *Journal of the Chemical Society, Faraday Transactions* **1991**, *87*, 2795–2804; bG. J. Millar, C. H. Rochester, K. C. Waugh, *Journal of the Chemical Society, Faraday Transactions* **1992**, *88*, 2257–2261.

[7]	R. Zou, M. Liu, C. Shen, K. Sun, C.-J. Liu, *Chemical Communications* **2024**, *60*, 1872–1875.

[8]	aG. J. Millar, D. Newton, G. A. Bowmaker, R. P. Cooney, *Appl. Spectrosc.* **1994**, *48*, 827–832; bG. J. Millar, C. H. Rochester, K. C. Waugh, *Journal of the Chemical Society, Faraday Transactions* **1991**, *87*, 2785–2793; cG. J. Millar, C. H. Rochester, K. C. Waugh, *Journal of Catalysis* **1995**, *155*, 52–58.

[9]	A. J. Al Abdulghani, S. Ganguly, R. H. Hagmann, Z. Sun, M. Alvear, L. O. Mark, E. Nikolla, Y. J. Pagán-Torres, I. Hermans, *Journal of the American Chemical Society* **2025**, *147*, 27438–27448.

[10]	M. Calatayud, S. E. Collins, M. A. Baltanás, A. L. Bonivardi, *Physical Chemistry Chemical Physics* **2009**, *11*, 1397–1405.

[11]	S. G. Neophytides, A. J. Marchi, G. F. Froment, *Applied Catalysis A: General* **1992**, *86*, 45–64.

[12]	aA. de Juan, J. Jaumot, R. Tauler, *Analytical Methods* **2014**, *6*, 4964–4976; bJ. Jaumot, A. de Juan, R. Tauler, *Chemometrics and Intelligent Laboratory Systems* **2015**, *140*, 1–12.
